# Comprehensive profiling of novel epithelial–mesenchymal transition mediators and their clinical significance in colorectal cancer

**DOI:** 10.1038/s41598-021-91102-9

**Published:** 2021-06-03

**Authors:** Satoshi Ishikawa, Naohiro Nishida, Shiki Fujino, Takayuki Ogino, Hidekazu Takahashi, Norikatsu Miyoshi, Mamoru Uemura, Taroh Satoh, Hirofumi Yamamoto, Tsunekazu Mizushima, Yuichiro Doki, Hidetoshi Eguchi

**Affiliations:** 1 Department of Gastroenterological Surgery, Osaka University Graduate School of Medicine, 2-2 Yamadaoka, Suita, Osaka 565-0871 Japan; 2 Department of Frontier Science for Cancer and Chemotherapy, Osaka University Graduate School of Medicine, 2-2 Yamadaoka, Suita, Osaka 565-0871 Japan

**Keywords:** Colorectal cancer, Oncogenes

## Abstract

Epithelial–mesenchymal transition (EMT) is a drastic phenotypic change during cancer metastasis and is one of the most important hallmarks of aggressive cancer. Although the overexpression of some specific transcription factors explains the functional alteration of EMT-induced cells, a complete picture of this biological process is yet to be elucidated. To comprehensively profile EMT-related genes in colorectal cancer, we quantified the EMT induction ability of each gene according to its similarity to the cancer stromal gene signature and termed it “mesenchymal score.” This bioinformatic approach successfully identified 90 candidate EMT mediators, which are strongly predictive of survival in clinical samples. Among these candidates, we discovered that the neuronal gene ARC, possibly originating from the retrotransposon, unexpectedly plays a crucial role in EMT induction. Profiling of novel EMT mediators we demonstrated here may help understand the complexity of the EMT program and open up new avenues for therapeutic intervention in colorectal cancer.

## Introduction

Epithelial–mesenchymal transition (EMT) is a phenotypic change of epithelial cells, in which cells lose epithelial features such as cell adhesion ability and polarity, and acquire motility and invasive capacity^1,2^. EMT plays a crucial role in human organ development and wound healing, and also potently contributes to tumorigenesis, invasion, metastasis and chemoresistance in human malignancies^3–5^.Given that more than half of the patients with colorectal cancer (CRC) develop liver metastases during their lifetime, and two-thirds of them have a fatal outcome, overcoming EMT might be an effective strategy to improve the prognosis of patients with CRC^6,7^.

Well-established transcription factors such as Zinc Finger E-Box Binding Homeobox 1/2 (ZEB1/2), Snail Family Transcriptional Repressor 1/2 (SNAI1/2), and Twist Family BHLH Transcription Factor 1 (TWIST1) execute EMT in response to signaling factors, including transforming growth factor beta (TGF-β)^8–12^. These transcription factors directly or indirectly repress the expression of Cadherin 1 (CDH1), which is essential for maintaining the epithelial structure^13^. In contrast, they promote the expressions of mesenchymal genes, including Vimentin (VIM) and Cadherin 2 (CDH2)^14^. Vimentin is a type III intermediate filament protein expressed mainly in mesenchymal cells and forms the cytoskeleton^15^, and Cadherin 2, also known as N-cadherin, is a calcium-dependent cell adhesion molecule that can be involved in both cell–cell adhesion and the migration of fibroblasts and mesenchymal cells, depending on the cellular context^16^. EMT-inducing transcription factors are post-transcriptionally regulated by non-coding RNAs, represented by the miR-200 family^17^. Several epigenetic and post-translational modifications such as methylation, acetylation, and phosphorylation, also contribute to the EMT program^18,19^. Despite these key findings, it is still challenging to comprehensively identify EMT-inducing genes because of the difficulty in detecting EMT-induced cells in clinical samples. Moreover, it has recently been reported that EMT is not a simple epithelial-stromal biphasic change but involves continuous intermediate states, between which cells move back and forth^14,20,21^. This finding is significant and intriguing for understanding the role of EMT in tumor dissemination and metastasis, whereas it complicates the concept of EMT and makes analyses of EMT-related genes difficult.

A major obstacle in the study of EMT is in the identification of EMT-induced cells in vivo^22^. EMT-induced cells lose their epithelial characteristics and acquire stromal characteristics. Therefore, distinguishing the gene signature of EMT-induced cancer cells from that of stromal cells in a tumor using traditional genetic and molecular analyses of bulk samples is a difficult task^23^. In 2015, two reports elegantly demonstrated that stromal tissue is the primary origin of the EMT signature in CRC bulk samples and that what we considered to be gene expression profiles of EMT-induced cancer cells might be that of contaminated stromal components^24,25^. This finding led us to the idea that cancer epithelium and stromal gene expression profiles need to be separately analyzed. Independent transcriptome analysis of cancer epithelium and stroma was performed using our previously developed laser micro-dissected CRC samples, and we combined these data with a multi-layered bulk sample dataset from The Cancer Genome Atlas (TCGA). The analysis was successful in identifying candidates of EMT mediators, most of which were unexplored as EMT-related genes. Among these candidates, we discovered that the neuronal gene ARC (activity regulated cytoskeleton associated protein) plays a crucial role in EMT regulation. Although ARC has been studied specifically in neuroscience, the association between ARC and human malignancy has been reported in detail for the first time.

Here, we propose a new method to comprehensively identify EMT related genes and evaluate the functional importance and the clinical significance of these genes.

## Results

### Extraction of candidate EMT-related genes

**Figure 1:**
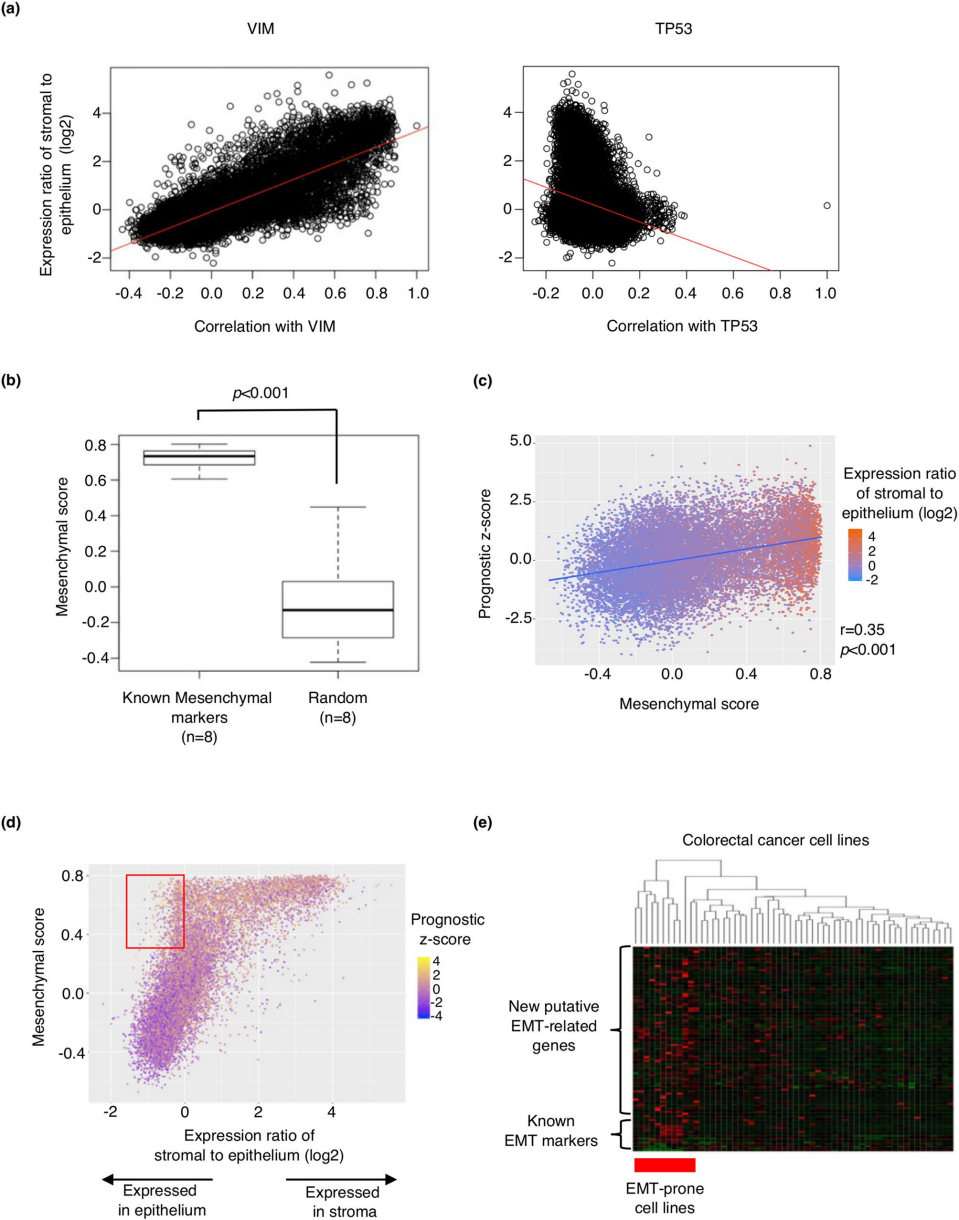
Identification of novel candidate EMT-related genes. (**a**) Calculation of “mesenchymal scores.” The horizontal axis represents Pearson’s correlation coefficients between two target genes and all other genes in CRC in TCGA dataset, and the vertical axis represents log fold change in the expression of all genes in the cancer stroma to epithelium in GSE35602. The left plot shows VIM as the target gene, whereas the right plot shows TP53 as the target gene. There was a significant correlation in VIM (r = 0.80, *p* < 0.001) but no correlation in TP53. We termed the correlation coefficients as “mesenchymal scores.” (**b**) Mesenchymal scores in known mesenchymal markers and randomly selected genes, *p* < 0.001. Known mesenchymal markers: VIM, SNAI2, ZEB1, ZEB2, TWIST1, CDH2, TGFB1, and FOXC2. (**c**) Correlation between z-scores of prognosis and mesenchymal scores. Z-scores of prognosis and mesenchymal scores of all genes were plotted in two dimensions (r = 0.35, *p* < 0.001). Red points indicate genes with high log fold change in the expression of all gene in the cancer stroma to epithelium, whereas blue points indicate genes with low log fold change. (**d**) Extraction of new putative EMT-related genes. The horizontal axis represents log fold change in the expression of all genes of the cancer stroma to epithelium, and the vertical axis represents mesenchymal scores. Colors of points indicate z-scores of prognosis. The red frame includes genes with mesenchymal scores > 0.3, the expression ratio in stroma to epithelium (log2) < 0, and z-scores of prognosis > 1.96 as candidates for novel EMT-related genes. (**e**) Unsupervised hierarchical clustering of CRC cell lines performed using expression data of the known EMT-related genes and new putative EMT-related genes from the CCLE dataset. *EMT* epithelial–mesenchymal transition, *CRC* colorectal cancer, *TCGA* The Cancer Genome Atlas, *CCLE* Cancer Cell Line Encyclopedia.

To comprehensively identify EMT-related genes, we started with the quantification of the EMT induction ability of each gene. In Fig. 1a, the horizontal axis represents the Pearson’s correlation coefficient between the target gene and all other genes in TCGA dataset, whereas the vertical axis represents the log fold change in the expression levels of all genes in the cancer stroma to epithelium in our own developed dataset (Fig. 1a, VIM and TP53 as examples). In this plot, we assumed that if a target gene was associated with EMT function, its neighboring genes (right side in the horizontal axis in Fig. 1a) should have an abundant mesenchymal gene signature and a high expression ratio of stroma to epithelium (see “Methods” for details). Indeed, the representative mesenchymal marker VIM showed a high correlation coefficient (r = 0.80, *p* < 0.001) (Fig. 1a, left), while Tumor Protein P53 (TP53) had no correlation (Fig. 1a, right). TP53 protects the genome from changes that lead to tumorigenesis^26^, and its mutations are known to be involved in various types of malignancies but not directly related to EMT. We termed this correlation coefficient “Mesenchymal score” because this value represents how the target and its neighboring genes are associated with the mesenchymal gene expression signature. Mesenchymal scores were significantly higher for eight known mesenchymal markers (VIM, SNAI2, ZEB1, ZEB2, TWIST1, CDH2, Transforming Growth Factor Beta 1 (TGFB1), and Forkhead Box C2 (FOXC2)) than eight randomly selected genes (*p* < 0.001) (Fig. 1b). Genes associated with immune response also exhibited high mesenchymal scores (Supplementary Fig. 2a, b, Supplementary Text 2). Immune-related genes were derived from published gene signatures as referenced^27^. Importantly, mesenchymal scores were significantly associated with poor prognosis in CRC (r = 0.35, *p* < 0.001) (Fig. 1c). Although genes preferably expressed in stromal tissue tended to have high mesenchymal scores, we found a subset of genes dominantly expressed in the cancer epithelium despite their high mesenchymal scores (red framed in Fig. 1d). We focused on this subpopulation because genes preferably expressed in the cancer epithelium with high mesenchymal scores could be upstream molecules in the EMT pathway and may have an active function. We extracted 90 highly expressed genes in the epithelium with high mesenchymal scores and z-scores as candidates of EMT-related genes (Fig. 1d, see “Methods” for details). The location of the eight known mesenchymal genes in Fig. 1d are shown in Supplementary Fig. 1.

### Validation of the candidate EMT-related genes in CRC cell lines

Next, we validated the expression status of the candidate EMT-related genes in the Cancer Cell Line Encyclopedia (CCLE) database^28^ to examine their expression in cancer cells and not in stroma. We first defined EMT-prone cell lines (MDST8, HS675T, HS698T, HS255T, SW480, NCIH716, RKO, COLO320, and SW620) by unsupervised hierarchical clustering using known EMT-related genes (Supplementary Fig. 3) and then analyzed the expression status of the candidate genes in these cell lines. As a result, the candidate genes as well as the established mesenchymal markers were highly expressed in the EMT-prone cell lines, indicating the significance of the newly identified genes as EMT-related genes in CRC cells (Fig. 1e).

### Association between candidate genes and EMT

**Figure 2:**
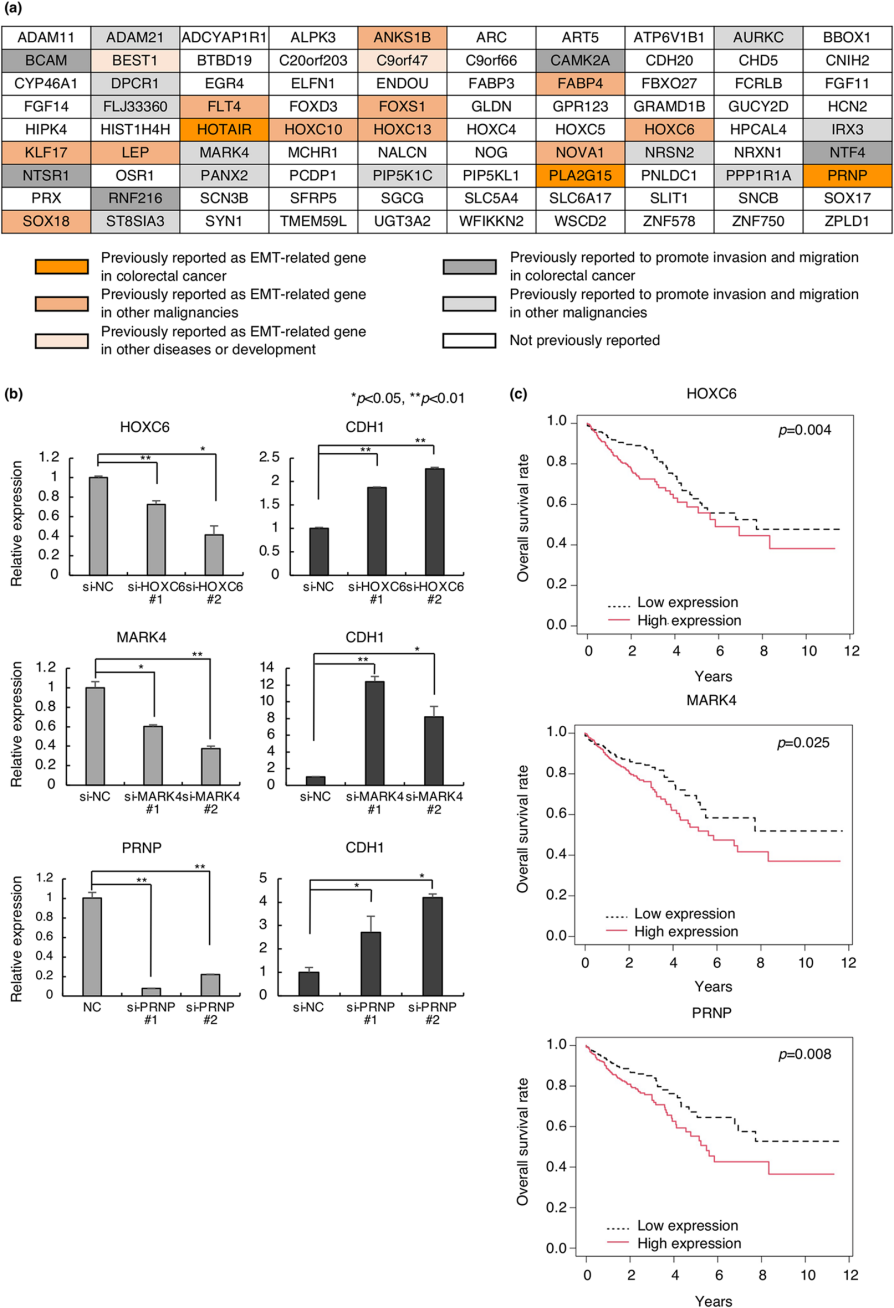
Association between candidate genes and EMT. (**a**) Previous reports of 90 candidate genes of EMT, cell invasion, or migration. Genes previously reported as EMT-related genes in CRC are shown in red, genes in other malignancies are in orange, and genes in other disease and development are in light orange. Genes previously reported to promote invasion and migration in CRC are shown in dark gray, genes in other malignancies are in light gray, and genes with no previous reports associated with either EMT, invasion, or migration are in white. (**b**) Alteration of CDH1 expression by knockdown of three candidate genes, HOXC6, MARK4, and PRNP in HCT116. All experiments were conducted in triplicate. Error bars show standard errors of the mean. Asterisks denote significant differences using unpaired 2-tail t-test (*p < 0.05, **p < 0.01). (**c**) Kaplan–Meier curves for overall survival in patients with CRC according to expression of HOXC6, MARK4, and PRNP (*p* = 0.004, 0.025, and 0.008, respectively). Patients are divided into two groups by the mean. These data were obtained from Broad GDAC Firehose colorectal adenocarcinoma (COADREAD) dataset (https://gdac.broadinstitute.org/) (n = 615). *EMT* epithelial–mesenchymal transition, *CRC* colorectal cancer, *Si* small interfering, *NC* negative control.

We identified three genes in CRC and eleven genes in other malignancies with previous reports on EMT among the 90 candidates (Fig. 2a, Supplementary Text 1). For example, homeobox C6 (HOXC6), one of the candidate genes, contributes to invasion by inducing the EMT pathway in hepatocellular carcinoma, oral squamous cell carcinoma, and cervical cancer^29–31^. Microtubule affinity-regulating kinase 4 (MARK4) acts as a negative regulator of Hippo kinase, and abrogation of MARK4 attenuates cell growth and migration in breast cancer cells^32^. Prion protein (PRNP) is involved in tumors, including glioblastoma, breast cancer, prostate cancer, gastric cancer, and CRC^33^. Particularly in CRC, PRNP has been reported to lead to EMT via the extracellular signal-regulated kinase 2 pathway^34^. In fact, by silencing HOXC6, MARK4, and PRNP with siRNAs, an increase in CDH1 expression and a decrease in expression of some mesenchymal markers were observed in HCT116 cells (Fig. 2b and Supplementary Fig. 4a, b, c). In DLD1 cells, knockdown of HOXC6 and MARK4 increased CDH1 expression, whereas knockdown of PRNP did not give consistent results. These results indicate that these genes are not just downstream molecules in the EMT pathway. Importantly, high expression of these three genes was associated with poor prognosis in CRC patients in TCGA database (*p* = 0.004, *p* = 0.025, and *p* = 0.008, respectively) (Fig. 2c). Thus, the candidate genes include several previously reported EMT regulators, but most of them are unexplored as EMT-related genes (Fig. 2a).

### The neuronal gene ARC is involved in EMT in colorectal cancer

**Figure 3:**
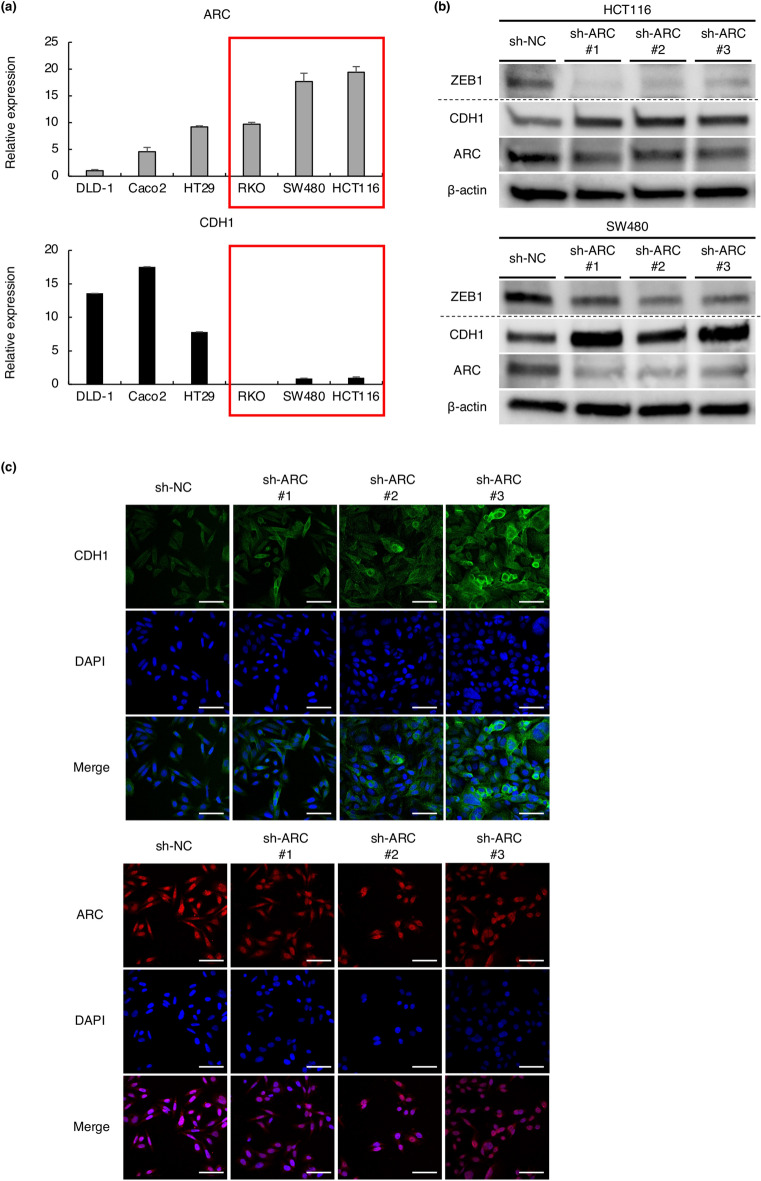
Association between the neuronal gene ARC and EMT. (**a**) ARC and CDH1 expression in six CRC cell lines (DLD-1, Caco2, HT29, RKO, SW480, and HCT116). Red boxes indicate cell lines with relatively high expression of ARC. All experiments were conducted in triplicate. Error bars show standard errors of the mean. (**b**) Alteration of CDH1 and ZEB1 expression by knockdown of ARC using shRNAs in western blot analysis. Results of ZEB1 were shown from different membranes using the same samples. Quantification of blots and full-length blots are presented in Supplementary Fig. 6b, c and Supplementary Fig. 11a, respectively. (**c**) Immunocytochemistry of CDH1 and ARC in SW480 cells expressing sh-NC and sh-ARC. Experiments were conducted in triplicate. Scale bars indicate 50 µm. *EMT* epithelial–mesenchymal transition, *CRC* colorectal cancer, *Sh* short hairpin, *NC* negative control.

The neuronal gene ARC has no previous reports about EMT or malignancies, has an association with prognosis or stage, and has a high mesenchymal score among the candidate genes. As such, we focused on ARC as a novel candidate as an EMT mediator. We investigated ARC expression in six CRC cell lines, including DLD-1, Caco2, HT29, RKO, SW480, and HCT116, and found that ARC and CDH1 expression showed an inverse correlation trend (Fig. 3a). The mesenchymal markers tended to be highly expressed in cell lines with high ARC expression, although there were considerable differences in the expression of mesenchymal markers among the cell lines (Supplementary Fig. 5). We established stable cell lines with short hairpin RNAs (shRNAs) to ARC in HCT116, SW480, and DLD1 cells and used them in the following experiments. Western blotting was performed to examine the alteration in representative epithelial and mesenchymal markers due to knockdown of ARC. The results showed that knockdown of ARC upregulates CDH1 expression and downregulates expression of ZEB1 and some other mesenchymal markers in HCT116 and SW480. (Fig. 3b, Supplementary Fig. 6a, b, c). Immunocytochemistry also showed that knockdown of ARC upregulated the expression of CDH1, which is mainly expressed on the cell membrane, but did not clearly alter the localization of ARC (Fig. 3c).

In addition, we established ARC overexpressed cells in DLD1. In contrast, overexpression of ARC decreased CDH1 expression (Supplementary Fig. 8a).

### ARC Knockdown sensitizes CRC cells to oxaliplatin and suppresses cell migration and invasion

**Figure 4:**
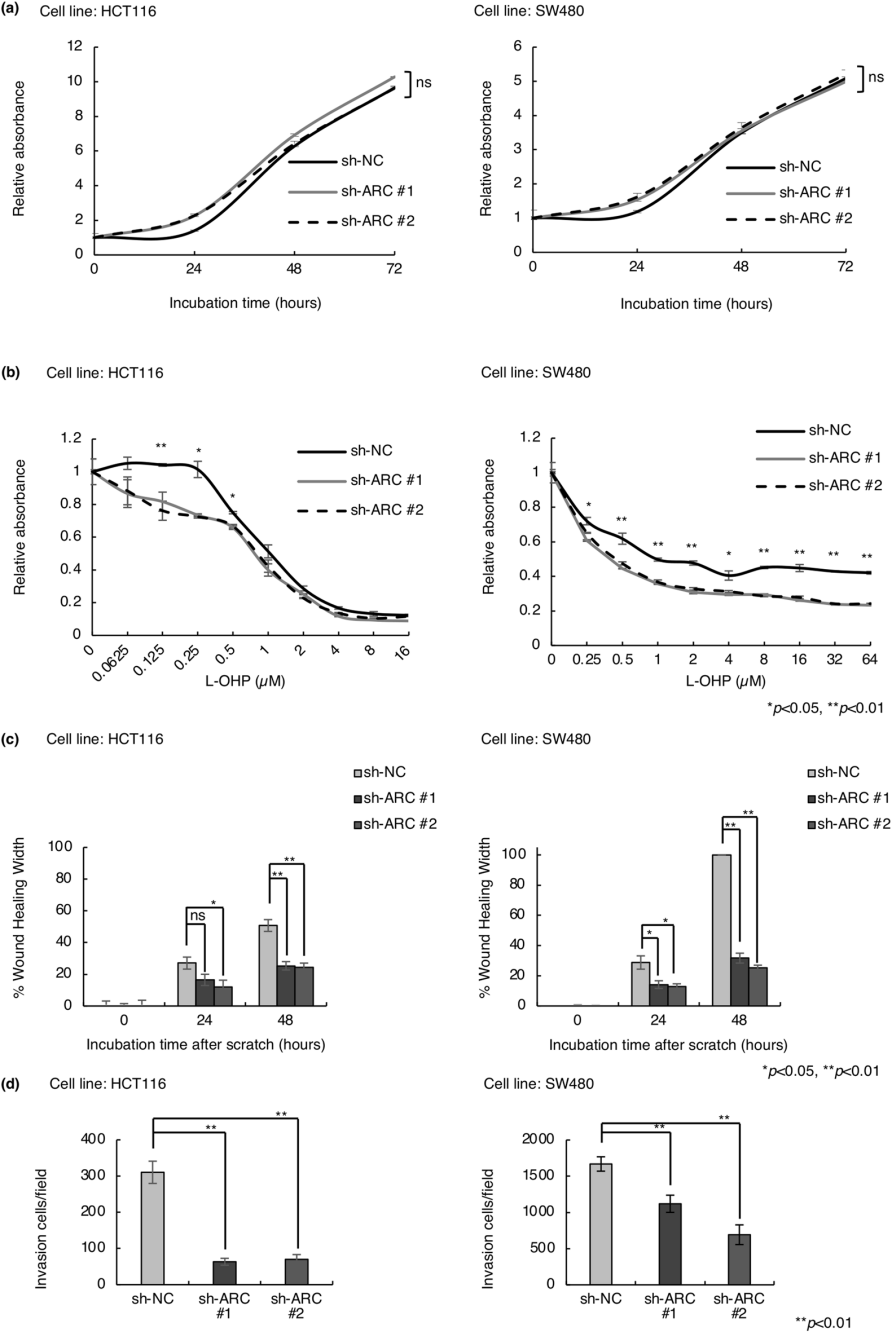
Alteration of cell proliferation, drug sensitivity, migration capacity, and invasion capacity by knockdown of ARC. (**a**) Cell proliferation assay in HCT116 and SW480 cells expressing sh-NC and sh-ARC. (**b**) Chemosensitivity assay to L-OHP in HCT116 and SW480 cells expressing sh-NC and sh-ARC. (**c**) Scratch wound healing assay in HCT116 and SW480 cells expressing sh-NC and sh-ARC. (**d**) Cell invasion assay in HCT116 and SW480 cells expressing sh-NC and sh-ARC. All experiments were conducted in triplicate. Error bars represent standard errors of the mean. Asterisks denote significant differences using the unpaired 2-tail t-test (*p < 0.05, **p < 0.01). *Sh* short hairpin, *NC* negative control, *L-OHP* oxaliplatin.

Previous reports have shown that cells undergoing EMT exhibited reduced proliferation and increased migration, invasion, and chemoresistance^21^. Cell proliferation was not altered in HCT116 and SW480 cells expressing sh-ARC (Fig. 4a). On the other hand, chemosensitivity to oxaliplatin was significantly increased (Fig. 4b). Moreover, ARC knockdown suppressed cell migration and invasion capacity in HCT116 and SW480 cells (Fig. 4c,d, Supplementary Fig. 7a,b). In contrast, overexpression of ARC decreased chemosensitivity to L-OHP and promote cell migration and invasion in DLD1 cells (Supplementary Fig. 8b,c,d).

### ARC expression is associated with the TGF-β pathway

**Figure 5:**
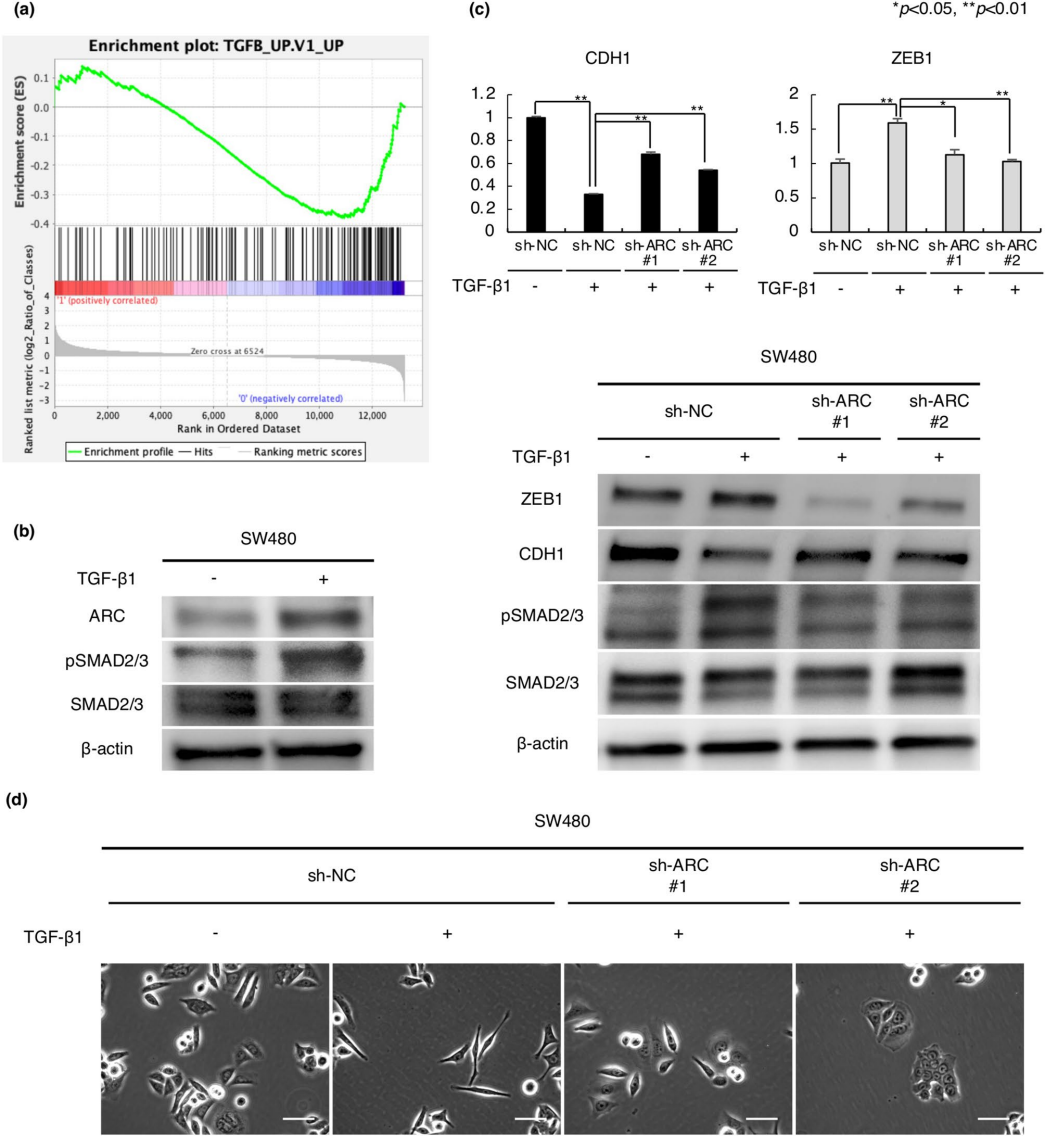
Involvement of ARC in TGF-β-mediated EMT pathway. (**a**) Association between ARC expression and upregulated genes in a panel of epithelial cell lines by TGF-β1 in GSEA (NES = − 1.499, *q* = 0.130). (**b**) Alteration of ARC expression in response to TGF-β1 in western blot analysis. Full-length blots are presented in Supplementary Fig. 11d. (**c**) Alteration of CDH1 expression in sh-NC and sh-ARC SW480 cell lines in response to TGF-β1 in qPCR and alteration of CDH1 and ZEB1 expression in sh-NC and sh-ARC SW480 cells in response to TGF-β1 in western blot analysis. Error bars show standard errors of the mean. Asterisks denote significant difference using unpaired 2-tail t-test (*p < 0.05, **p < 0.01). Full-length blots are presented in Supplementary Fig. 1^11^. (d) TGF-β1-induced change in cell shape in sh-NC and sh-ARC SW480 cell lines. Scale bars indicate 50 µm. Experiments were conducted in triplicate. *TGF* transforming growth factor, *EMT* epithelial–mesenchymal transition, *GSEA* gene set enrichment analysis, *NES* normalized enrichment score, *sh* short hairpin, *NC* negative control, *qPCR* quantitative polymerase chain reaction.

We performed RNA sequencing of sh-negative control (sh-NC) and sh-ARC #1 and analyzed the results using GSEA. GSEA showed that ARC expression was associated with genes upregulated in a panel of epithelial cell lines by TGF-β1 (‘TGFB_UP.V1_UP’; https://www.gsea-msigdb.org/gsea/msigdb/cards/TGFB_UP.V1_UP.html) with normalized enrichment scores of -1.499 and a false discovery rate of 0.130, as shown in Fig. 5a. To investigate the effect of activation of TGF-β pathway on ARC expression, we added TGF-β1 to SW480 and DLD1 cells. ARC expression was induced by exposure of TGF-β1 (Fig. 5b, Supplementary Fig. 9a, b). Furthermore, the effect of TGF-β1 on suppression of CDH1 expression and upregulation of expression of some mesenchymal markers was attenuated in sh-ARC cells (Fig. 5c, Supplementary Fig. 9c, d). The TGF-β1-induced change in cell shape into the spindle shape was also attenuated in sh-ARC cells (Fig. 5d, Supplementary Fig. 9e). On the other hand, TGF-β1 strongly inhibited cell proliferation, and this effect was not altered significantly by ARC knockdown (Supplementary Fig. 9f.).

### ARC expression predicts clinical outcomes in patients with CRC

**Figure 6:**
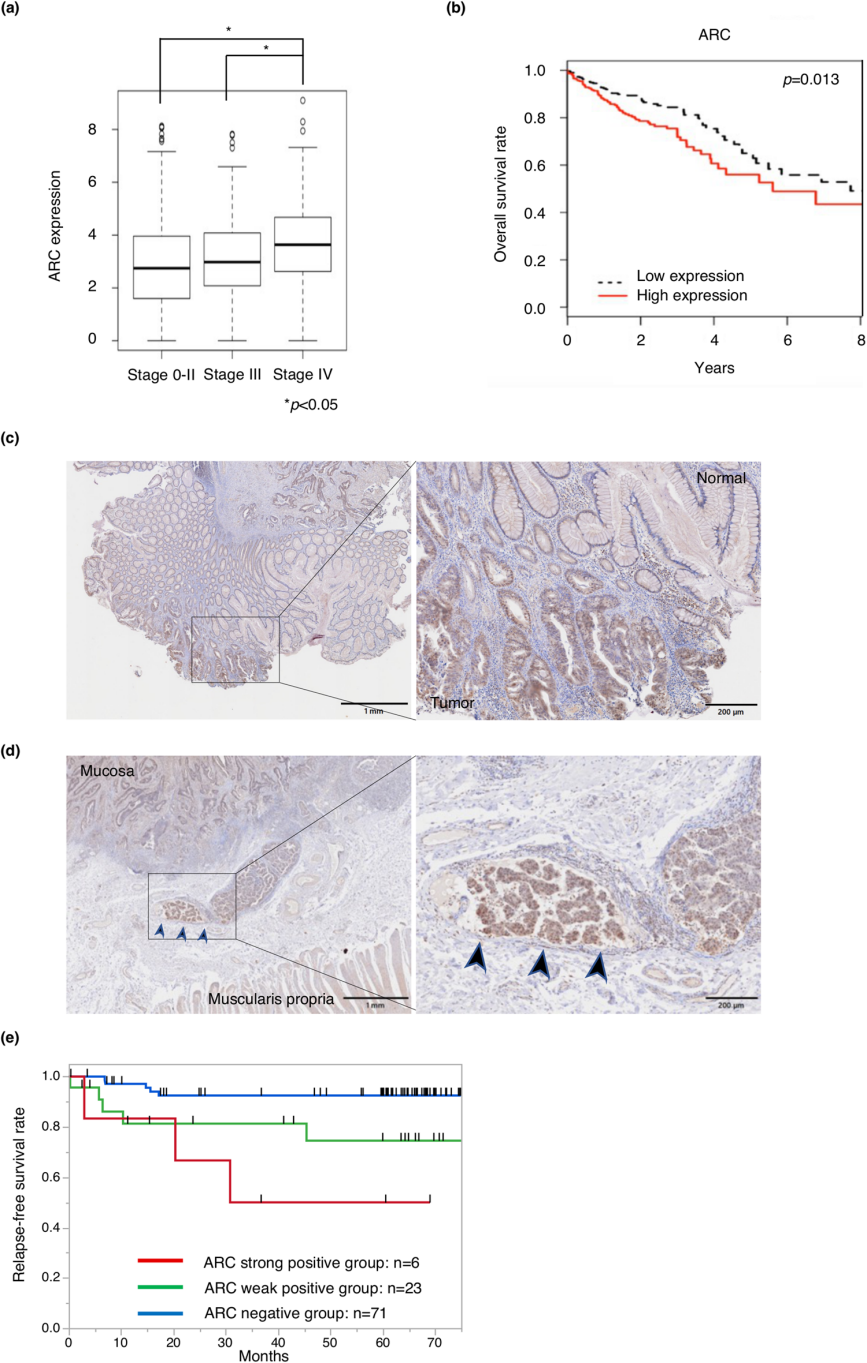
ARC expression in clinical samples in patients with CRC. (**a**) Box plot showing correlation between ARC expression and disease stage in patients with CRC in TCGA dataset. Stage 0–II: n = 271; stage III: n = 133; stage IV: n = 62. Within all samples, we analyzed samples which had complete TNM clinical stage information. Asterisks denote significant difference (*p < 0.05). (**b**) Kaplan–Meier curves for overall survival in patients with CRC according to expression of ARC (*p* = 0.013). Patients are divided into two groups by the median. These data were obtained from Broad GDAC Firehose colorectal adenocarcinoma (COADREAD) dataset (https://gdac.broadinstitute.org/) (n = 615). (**c**) IHC of ARC in tumor and normal tissue in a specimen of CRC. Scale bars indicate 1 mm (left) and 200 µm (right). (**d**) IHC of ARC in the deepest parts of tumor in a specimen of CRC. Scale bars indicate 1 mm (left) and 200 µm (right). (**e**) Kaplan–Meier curves for relapse-free survival in patients with CRC according to ARC expression. Patients are divided into three groups by the intensity of ARC expression in IHC (n = 100). *CRC* colorectal cancer, *TCGA* The Cancer Genome Atlas, *IHC* immunohistochemical staining.

In TCGA dataset, ARC expression was correlated with disease stage, and the group with high ARC mRNA expression exhibited a poorer prognosis than the group with low expression (Fig. 6a,b). To investigate the association of ARC protein expression with clinicopathological factors and prognosis, we performed immunohistochemical staining (IHC) for ARC protein in patients with stage 0–III CRC. IHC showed that ARC was expressed more strongly in cancer tissues than in normal tissues, and was predominantly expressed in the cancer epithelium rather than in the stroma (Fig. 6c, Supplementary Fig. 10b). In addition, ARC was especially highly expressed in the invasive front of cancer (Fig. 6d, Supplementary Fig. 10c). We divided patients into three groups according to the intensity of ARC expression: strong, weak, and negative (Supplementary Fig. 10a). Patient characteristics were classified according to ARC expression (negative expression: negative group, weak and strong positive expression: positive group), as shown in Table 1 and Supplementary Table 1. The ARC-positive group exhibited more frequent lymphatic invasion than the negative group. The results for the univariate and multivariate analyses for relapse-free survival (RFS) are presented in Table 2. RFS was significantly related to elevated CEA levels, presence of lymph node metastasis, presence of venous invasion, and positive ARC expression. Of these, venous invasion and positive ARC expression were independent prognostic factors for RFS in the multivariate analysis. Figure 6e shows Kaplan–Meier curves according to the intensity of ARC expression. The ARC-positive group had a significantly shorter RFS than the negative group in the Kaplan–Meier analysis (*p* = 0.003) (Supplementary Fig. 10d). The ARC positive group tended to have worse overall survival (OS) than the negative group, although there was no statistically significant difference (*p* = 0.150) (Supplementary Fig. 10e). Moreover, IHC with an anti-E-cadherin antibody was performed, and tumors with low ARC expression tend to have high E-cadherin expression (Supplementary Fig. 10f., left) and vice versa (Supplementary Fig. 10f., right).

**Table 1:** Relationship between ARC expression and patient characteristics.

Variable	ARC expression		
	Negative (n = 71)	Positive (n = 29)	*p* value
Age^a^ (years)	66.3 ± 13.3	66.6 ± 11.1	0.997
**Sex**			0.043
Male	36 (63.2)	21 (36.8)	
Female	35 (81.4)	8 (18.6)	
**Primary tumor site**			0.842
Colon	50 (70.4)	21 (29.6)	
Rectum	21 (72.4)	8 (27.6)	
**Histological grade**			0.060
Tub1, Tub2	66 (69.8)	29 (30.2)	
Por, Muc	5 (100.0)	0 (0.0)	
**Tumor invasion**			0.238
Tis, T1, or T2	31 (77.5)	9 (22.5)	
T3 or T4	40 (66.7)	20 (33.3)	
**Lymph node metastasis**			**0.041**
Absent	52 (77.6)	15 (22.4)	
Present	19 (57.6)	14 (42.4)	
**Lymphatic invasion**			**0.005**
Absent	36 (85.7)	6 (14.3)	
Present	35 (60.3)	23 (39.7)	
**Venous invasion**			0.953
Absent	51 (70.8)	21 (29.2)	
Present	20 (71.4)	8 (28.6)	
**Pathological stage**			**0.041**
0, I, IIA, IIB, or IIC	52 (77.6)	15 (22.4)	
IIIA, IIIB, or IIIC	19 (57.6)	14 (42.4)	

Tub1, well-differentiated adenocarcinoma; Tub2, moderately differentiated adenocarcinoma; Por, poorly differentiated adenocarcinoma; Muc, mucinous adenocarcinoma.

Bold values indicate *p* < 0.05.

^a^Continuous variable.

**Table 2:** Univariate and multivariate analyses of relapse-free survival.

Variable	Univariate			Multivariate		
	HR	95% CI	*p* value	HR	95% CI	*p* value
Sex (male/female)	1.237	0.405–3.782	0.709			
Preoperative CEA (≥ 5/< 5)	5.810	1.748–19.31	**0.004**	2.484	0.662–9.314	0.177
Primary tumor site (rectum/colon)	0.746	0.205–2.713	0.657			
Tumor invasion (T3–4/Tis, T1–2)	2.510	0.691–9.124	0.162			
Lymph node metastasis (present/absent)	5.114	1.573–16.63	**0.007**	1.358	0.322–5.718	0.677
Lymphatic invasion (present/absent)	4.336	0.961–19.57	0.056			
Venous invasion (present/absent)	6.854	2.109–22.28	**0.001**	4.251	1.114–16.22	**0.034**
ARC expression (positive/negative)	4.677	1.528–14.31	**0.007**	5.306	1.366–20.61	**0.016**

*HR* hazard ratio, *CI* confidence interval, *CEA* carcinoembryonic antigen.

HR and 95%CI of histological grade cannot be calculated because none of the five patients with Por or Muc had cancer relapse.

Bold values indicate *p* < 0.05.

## Discussion

EMT was initially described as an essential biological process for early embryogenesis^35^, and developmental genetics have contributed to the identification of several transcription factors that organize EMT in embryonic development^36^. Subsequently, these genes have also been shown to play a vital role in cancer progression and metastasis. For example, core transcription factors, such as SNAI1/2, ZEB1/2, and TWIST1, have been discovered in embryology and diverted to cancer research^2^. Although in vitro models overexpressing these transcriptional factors recapitulate EMT-induced cancer cells, the gene regulatory network underlying EMT in human cancer tissues is largely unknown. There are diverse EMT programs in cancer; therefore, the EMT program in cancer cannot be precisely defined by limited markers alone that were originally discovered in development.

Conventional gene expression profiling has mainly been performed on bulk tissue samples containing an abundant amount of stromal tissues, which may influence the gene expression signature^23^. The gene signature of EMT-induced epithelial cells resembles that of stromal cells, and therefore, it is difficult to analyze the EMT program using bulk samples. In this study, using laser micro-dissected CRC samples, we profiled epithelial-specific gene expression and compared them to that of stromal tissues. By quantifying the EMT induction ability of each gene, we identified 90 candidates of EMT mediators, some of which have been previously reported as EMT-related genes in CRC or other malignancies. These candidates were strongly expressed in CRC cell lines with high expression of known mesenchymal markers, indicating that they are associated with EMT. Due to their prognostic relevance in patients with CRC in the TCGA database, HOXC6, MARK4, and PRNP were selected as examples of candidate genes. Knockdown experiments of the three genes enhanced CDH1 expression and partially downregulated mesenchymal gene expression in HCT116, suggesting that they are not just expression markers for the mesenchymal state but possibly act as upstream molecules in the EMT pathway. HOXC6 belongs to the homeobox family, members of which code for a highly conserved set of proteins that plays an essential role in morphogenesis^37^. Its involvement in EMT has been previously reported in hepatocellular carcinoma, oral squamous cell carcinoma, and cervical cancer^29–31^. Because morphogenesis is a key component of embryonic development, the involvement of the homeobox family in EMT induction is entirely reasonable. Another candidate, MARK4, belongs to the microtubule affinity-regulating kinase family that exhibits diverse functions, including embryonic development, asymmetric cell division, and cell polarity definition^38,39^. In cancer, this gene have been shown to inhibit the Hippo signaling pathway and is associated with the migration ability of breast cancer cells^32^. Its involvement in CDH1 regulation has been identified for the first time in this study.

Besides, the candidates included several genes related to tyrosine kinase receptor signaling. For example, FGF11 and FGF 14 are members of the fibroblast growth factor (FGF) family, which are ligands of FGF receptors (FGFR) and involved in tumor proliferation, migration, and invasion^40^. HOXC10 binds the promoter region of human epidermal growth factor (HER) 3 and activates the PI3K/AKT pathway^41^. Tyrosine kinase signaling, including FGFR, HER, and c-MET signaling, is known to be involved in EMT, as is TGF-β signaling^42^.

To find a novel EMT-related gene, we focused on the neuronal gene ARC because it has no previous report about EMT, has an association with prognosis or stage, and has a high mesenchymal score among the candidate genes. ARC is a regulator of synaptic plasticity highly expressed in cortical and hippocampal glutamatergic neurons and crucially involved in learning and memory formation^43^. ARC mRNA is rapidly transported to the postsynaptic dendrites of active synapses, where it is translated and regulates the synaptic strength by α-amino-3-hydroxy-5-methylisoxazole-4-propionate receptors endocytosis^44^. Although ARC has been studied in detail in neuroscience, to the best of our knowledge, there have been no reports describing the association between ARC and human malignancies. The present study showed that TGF-β exposure enhanced ARC expression and that its knockdown attenuated TGF-β-mediated EMT via ZEB1 in CRC cell lines. Recently, Pastuzyn et al. demonstrated that ARC is evolutionally derived from a vertebrate lineage of Ty3/gypsy retrotransposons, of which retroviruses are also ancestors^45^. Surprisingly, ARC produces retrovirus-like capsids (Gag proteins) that enclose ARC mRNA, which can function as a neurotransmitter^43^. Although we have not investigated the extracellular function of ARC in the cancer microenvironment, it might be an attractive hypothesis that ARC mRNA can be transferred to neighboring cancer cells, where it also functions as an EMT inducer.

The present study has some limitations. First, given that EMT occurs in only a part of cancer cells, it may be necessary to extract only cells with a strong tendency for EMT and perform single-cell analysis. However, the method to extract only EMT-prone cells has not been established so far. The present method, using open microarray data from the purified epithelium and stroma, is a convenient and efficient way to successfully demonstrate the possibility that a wider variety of genes are involved in EMT than previously expected. Further investigation is required to clarify the relationships among these genes in the EMT program. Second, this method only extracts genes more highly expressed in the epithelium than in the stroma to exclude genes expressed in the stroma but not involved in EMT (Fig. 1d). However, some genes are expressed in the epithelium and induce EMT while being more highly expressed in the stroma. As a result, genes essential for EMT such as ZEB1/2, SNAI1/2, and TWIST1 were not listed (Fig. 2a, Supplementary Fig. 1). This method also ignored genes expressed in mesenchymal cells and induce EMT in cancer epithelial cells via tumor microenvironment. Another kind of approach is required to explore these genes.

In summary, our comprehensive analysis detecting EMT-related genes have revealed that genes with various functions are involved in the EMT. Although a few transcription factors initiate the EMT program, highly diversified mediators, as we demonstrated here, are orchestrated to proceed with the process, some of which are essential for completing the program. In cancer treatment strategies, transcription factors are difficult to target because of their significant impact on normal tissues. Our newly identified EMT mediators could be promising therapeutic targets to prevent cancer metastasis through the inhibition of the gene regulatory network underlying EMT.

## Methods

### Mesenchymal scores

We defined these correlat GSE35602 includes gene expression data of pure epithelial and stromal tissue obtained using laser micro-dissection technique from 13 CRC specimens^46^. For transcriptome analysis, we used mRNA-seq data from Broad GDAC Firehose colorectal adenocarcinoma (COADREAD) dataset (https://gdac.broadinstitute.org/), whose clinical data is available (n = 615). The data was downloaded from http://firebrowse.org/?cohort = COADREAD & download_dialog = true. The expression ratio in stroma to epithelium of each gene from GSE35602 and correlation of each gene expression with a target gene derived from TCGA were plotted in two dimensions, and Pearson’s correlation coefficients were calculated (Fig. 1a). We used formula below to calculate expression ratio between stroma and epithelium (vertical axis in Fig. 1a).

Gene X expression in stromal tissue/Gene X expression in epithelial tissue.

We defined these correlation coefficients as "Mesenchymal scores" and compared mesenchymal scores of eight known mesenchymal markers (VIM, SNAI2, ZEB1, ZEB2, TWIST1, CDH2, TGFB1, and FOXC2) with eight randomly selected genes (Fig. 1b). The randomly selected genes are computationally selected genes using “sample” function of the R program. We have performed this calculation more than twenty times.

### Extraction of candidate genes that induce EMT

Z-scores measured in the CRC dataset from TCGA were used to assess statistical associations between each gene and patient prognosis (Fig. 1b). The Z-score is a measurement of statistical significance and represents the value's position in terms of its distance from the average when measured in standard deviation units. A positive z-score indicates an adverse prognostic association in the univariable Cox regression model, whereas a negative z-score indicates a favorable prognostic association used in the previous study^47^. Z-scores were calculated using "coxph" function from package "survival" in the R program. We extracted genes with mesenchymal scores > 0.3, expression ratio in stroma to epithelium (log2) < 0, and z-scores of prognosis > 1.96 as candidates for novel EMT mediators (Fig. 1d). Unsupervised hierarchical clustering of CRC cell lines was performed using expression data of the known EMT-related genes from the CCLE to identify EMT-prone CRC cell lines (Supplementary Fig. 3), in which we examined the expression status of candidate genes (Fig. 1e).

### Cell culture

Human CRC cell lines, namely DLD-1, Caco-2, HT-29, RKO, SW480, and HCT116 were purchased from the American Type Culture Collection (Manassas, VA, USA) and cultured in Dulbecco’s modified Eagle’s medium (DMEM) supplemented with 10% fetal bovine serum (FBS) at 37 °C under an atmosphere of 5% CO2 in a humidified incubator.

### RNA interference

HOXC6, MARK4, and PRNP-specific small interfering RNAs (siRNAs) and a negative control siRNA (si-NC) were synthesized by Sigma-Aldrich; Merck KGaA (Darmstadt, Germany). The siRNA sequences were the following:

- si-HOXC6 #1, 5′-UCCUACUUCACUAACCCUU[dT][dT]-3′;
- si-HOXC6 #2, 5′-CCUCAAUUCCACCGCCUAU[dT][dT]-3′;
- si-MARK4 #1, 5′-GCAUCAUGAAGGGCCUAAA[dT][dT]-3′
- si-MARK4 #2, 5′-CCAUCUACCUUGGGAUCAA[dT][dT]-3′;
- si-PRNP #1, 5′-GCGUCAAUAUCACAAUCAA[dT][dT]-3′;
- and si-PRNP #2, 5′-GCCUAUUACCAGAGAGGAU[dT][dT]-3′.

The siRNAs were transfected into HCT116 cells using Lipofectamine RNAiMax (Invitrogen; Thermo Fisher Scientific, Waltham, MA, USA) at a final concentration of 50 nM. RNA was extracted 48 h after transfection. We also obtained three lentiviral vectors containing short hairpin RNAs (shRNAs) directed to ARC and an empty vector (pLKO.1 puro) from the MISSION TRC-Hs1.0 library (Sigma-Aldrich; Merck KGaA). The four lentiviral vectors were co-transfected into 293FT cells with VSVG and PAX2 plasmids using Lipofectamine 3000 (Invitrogen; Thermo Fisher Scientific). The supernatant containing the lentivirus was collected 48 h after transfection and added to SW480 cells with 5 μM polybrene. Stable colonies were selected clonally with 5 μg/ml puromycin for 14 days, and sh-negative control (sh-NC), sh-ARC #1, #2, and #3 were established.

### Lentivirus vector construction and overexpression

The full length of human cDNA of ARC was amplified by PCR and genetically ligated into the CSII-CMV-MCS-IRES2-Bsd lentivirus vector (provided by Dr. Miyoshi, RIKEN-BRC, Japan)^48^. The sequence was confirmed by direct sequencing. The vector was transfected into 293FT cells with envelope and packaging plasmids using Lipofectamine 3000 reagent (Thermo Fisher Scientific) according to the manufacturer’s protocol. After 48 h incubation, the supernatant was filtered and used for virus transduction to target cells. Stable clones were obtained after antibiotic selection. The overexpression of genes was confirmed by Western blotting and qRT-PCR.

### Quantitative reverse transcription polymerase chain reaction (qRT-PCR)

Total RNA was extracted from cells using TRI reagent (Molecular Research Center, Cincinnati, OH, USA). Complementary DNA synthesis was carried out using a High Capacity RNA-to-cDNA Kit (Applied Biosystems; Thermo Fisher Scientific). Quantitative polymerase chain reaction (qPCR) was performed on a QuantStudio 7 system (Applied Biosystems; Thermo Fisher Scientific) using the Thunderbird SYBR quantitative PCR mix (Toyobo Life Science, Osaka, Japan). GAPDH was used as a reference gene. The primer sequences were the following: HOXC6, 5′-GGAGAATGTCGTGTTCAGTTCC-3′ (forward) and 5′-GCGATTGAGGTCTGTGTGTTATG-3′ (reverse); MARK4, 5′-GTCAACAGACTGTGAGAGCATC-3′ (forward) and 5′-GTGTATGGCTTCAACTCCTCAC-3′ (reverse); PRNP, 5′-AGACCGACGTTAAGATGATGGA-3′ (forward) and 5′-TGGTAATAGGCCTGAGATTCCC-3′ (reverse); CDH1, 5′-GAGGATTTTGAGCACGTGAAGA-3′ (forward) and 5′-TAGTTCGAGGTTCTGGTATGGG-3′ (reverse); ZEB1, 5′-CAGAGGATGACCTGCCAACA-3′ (forward) and 5′-GATTTCTTGCCCTTCCTTTCC-3′ (reverse); GAPDH 5′-AGCCACATCGCTCAGACAC-3′ (forward) and 5′-GCCCAATACGACCAAATCC-3′ (reverse).

### Western blot analysis

The total protein was extracted from cells using radioimmunoprecipitation assay lysis buffer with protease and phosphatase inhibitors. The protein samples were separated by sodium dodecyl sulfate–polyacrylamide gel electrophoresis and then transferred to polyvinylidene fluoride membranes. Membranes were blocked, incubated with primary antibodies overnight at 4 °C, and then with secondary antibodies for 1 h at 25 °C. Chemiluminescent detection was performed using the ECL Prime Western Blotting Detection Reagent (GE Healthcare, Little Chalfont, UK) on Image Quant LAS4000 (Fujifilm, Tokyo, Japan). The following antibodies were used: anti-ARC rabbit polyclonal antibody (16290-1-AP; Proteintech, Rosemont, IL, USA) at dilution of 1:500, anti-E-cadherin monoclonal rabbit antibody (#3195; Cell Signaling Technology (CST), Danvers, MA, USA) at dilution of 1:1000, anti-ZEB1 monoclonal rabbit antibody (#3396; CST) at dilution of 1:1000, anti-pSMAD2/3 monoclonal rabbit antibody (#8828; CST) at dilution of 1:1000, anti-SMAD2/3 monoclonal rabbit antibody (#8685; CST) at dilution of 1:1000, and anti-actin polyclonal rabbit antibody (A2066; Sigma-Aldrich; Merck KGaA) at a dilution of 1:2000.

### Immunocytochemistry

Cells were fixed in 4% paraformaldehyde, blocked and, permeabilized with a 5% bovine serum albumin (BSA) with 0.3% Triton X100. Then cells were incubated with primary antibodies overnight at 4 °C, and then with secondary antibodies for 1 h at 25 °C, followed by Prolong Glass Antifade Mountant with Nucblue Stain (Thermo Fisher Scientific). Samples were examined under FV1000 laser scanning confocal microscope (Olympus, Tokyo, Japan). The following antibodies were used: anti-ARC rabbit polyclonal antibody (16290-1-AP; Proteintech) at dilution of 1:200, anti-E-cadherin monoclonal rabbit antibody (#3195; CST) at dilution of 1:200, and anti-rabbit IgG (H + L) F(ab′)2 fragment Alexa Fluor 647 conjugate (#4414, CST) at dilution of dilution 1:1000.

### Scratch wound healing and cell invasion assays

Cells were grown to confluence in 6-well plates, scratched with a 200 µL sterile pipette tip, and incubated in DMEM with 1% FBS. The distances between the wound edges were measured at 10 random sites in each well. Cell invasion assay was performed using the 24-well Corning BioCoat Matrigel Invasion Chambers (Corning, Tewksbury, MA, USA) according to the manufacturers’ instruction. The lower chambers were filled with DMEM with 10% FBS as a chemoattractant and 5 × 10^4^ cells suspended in DMEM with 1% FBS were seeded into the insert chambers. After incubation for 48 h, the invading cells were fixed and stained using a Diff-Quick Stain Kit (Sysmex, Kobe, Japan) and counted in five high-power fields.

### Cell proliferation assay

Cells were plated at a density of 4.0 × 10^3^ cells/well into 96-well plates and incubated. The number of living cells was evaluated using [2-(2-methoxy-4-nitrophenyl)-3-(4-nitrophenyl)-5-(2,4-disulfophenyl)-2H-tetrazolium] monosodium salt (Cell Counting kit-8; Dojindo Molecular Technologies, Kumamoto, Japan) after 24, 48, and 72 h.

### Chemosensitivity assay

In 96-well plates, 4 × 10^3^ cells were plated and incubated for 24 h. Then, the cells were exposed to various concentrations of oxaliplatin for 72 h, and cell viability was evaluated using Cell Counting kit-8 (Dojindo Molecular Technologies).

### RNA sequencing and gene set enrichment analysis

Total RNA isolated from sh-NC and sh-ARC #1 of SW480 was submitted to the NGS core facility of the Genome Information Research Center at the Research Institute for Microbial Diseases of Osaka University for RNA sequencing. Gene set enrichment analysis (GSEA) v. 4.0.3. was downloaded from the Broad Institute website (www.broadinstitute.org/gsea/index.jsp), and GSEA was performed using RNA sequencing data from sh-NC and sh-ARC #1 of SW480.

### EMT induction by TGF-β1

Cells were incubated in DMEM with 2.5 ng/ml recombinant human TGF-β1 (Peprotech, Rocky Hill, NJ, USA), 10 ng/ml recombinant human epidermal growth factor (Sigma-Aldrich, Saint Louis, MO, USA), 100 × Insulin-Transferrin-Selenium (Gibco; Thermo Fisher Scientific), and 50 nmol/l hydrocortisone for 72 h, after which RNA and protein were extracted.

### Clinical samples

A total of 144 consecutive patients with stage 0-III CRC who underwent curative resection at the Department of Gastroenterological Surgery, Osaka University, in 2013 were included. Clinical specimens were collected from 100 patients, excluding 11 patients with other synchronous cancers, 14 patients after endoscopic resection, 2 with inflammatory bowel disease, 1 who achieved a complete response to preoperative treatment, and 16 whose specimens were not available. The clinicopathological findings were classified based on the eighth edition of the Unio Internationalis Contra Cancrum (UICC) TNM classification. Postoperative patients underwent CT scans, blood examinations for serum CEA and CA19-9 levels every 3–6 months, and annual or biannual colonoscopies under the Japanese national guidelines^49^. Data regarding patient survival and recurrence were collected from medical records to evaluate OS and RFS. The Institutional Review Boards of Osaka University granted ethical approval for this study (approval ID: 08,226). All patients provided written informed consent. We confirm that all methods were carried out in accordance with approved guidelines and regulations of Osaka University.

### Immunohistochemical staining

The expression levels of ARC proteins were evaluated by IHC. All specimens were fixed in 10% buffered formalin and embedded in paraffin. The 3.5 μm thick sections were subjected to antigen retrieval for 20 min at 110 °C in 10 mM citrate buffer at pH 6.0, and the endogenous peroxidase activity was blocked with methanol supplemented with hydrogen peroxide. Sections were blocked by goat serum, incubated with the anti-ARC rabbit polyclonal antibody (16290-1-AP, Proteintech) at a dilution of 1:200 and anti-E-cadherin monoclonal rabbit antibody (#3195; CST) at dilution of 1:400, overnight at 4 °C, and then incubated with the secondary antibody at a 1:200 dilution at 25 °C for 30 min using VECTASTAIN Elite ABC Rabbit Immunoglobulin G kit (Vector Laboratories, Burlingame, CA, USA). We used human brain tissue as a positive control and assigned the specimen with the same intensity of staining as the positive control to the ARC strong group, whereas the unstained specimen was assigned to the ARC negative group. We assigned the specimen stained weaker than the positive control to the ARC weak positive group (Supplementary Fig. 10a).

### Statistical analysis

Measurement of mesenchymal scores and prognostic z-scores and clustering analysis were performed using the R software program, v. 3.5.0: Bioconductor package (R Core Team (2020). R: A language and environment for statistical computing. R Foundation for Statistical Computing, Vienna, Austria. URL https://www.R-project.org/). We used RNA sequencing by expectation maximization values (RSEM) for transcriptome analysis, which was one of the methods ﻿for quantifying transcript abundances from RNA-sequencing data^50^. Major pipelines including Firehose (https://gdac.broadinstitute.org/) use RSEM for quantification of gene expression. Experiments were conducted in triplicate, and data are presented as the mean ± standard error. The Student’s t-test was used to verify differences between the two groups in vitro. Patient characteristics are presented as the number of patients (percentage), and a continuous non-parametric variable was analyzed with the Mann–Whitney U-test and categorical variables with the chi-square test. Univariate and multivariate analyses were performed using a Cox proportional hazards model using JMP® software version 14 (SAS, Cary, NC, USA). All graphs in the main and supplementary figures were drawn using R, Microsoft Excel^®^, v. 16.0 (Microsoft Corporation (2018). Microsoft Excel, Redmond, WA, USA. URL https://office.microsoft.com/excel), or JMP.

## Supplementary Information

Supplementary Information.

## References

[1] Thiery JP, Acloque H, Huang RYJ, Nieto MA (2009). Epithelial–mesenchymal transitions in development and disease. Cell.

[2] Yang J (2020). Guidelines and definitions for research on epithelial–mesenchymal transition. Nat. Rev. Mol. Cell Biol..

[3] Brabletz T (2012). To differentiate or not—Routes towards metastasis. Nat. Rev. Cancer.

[4] Kalluri R, Weinberg RA (2009). The basics of epithelial–mesenchymal transition. J. Clin. Investig..

[5] Pastushenko I (2018). Identification of the tumour transition states occurring during EMT. Nature.

[6] Vu T, Datta P (2017). Regulation of EMT in colorectal cancer: A culprit in metastasis. Cancers.

[7] Zarour LR (2017). Colorectal cancer liver metastasis: Evolving paradigms and future directions. Cell. Mol. Gastroenterol. Hepatol..

[8] Park S-M, Gaur AB, Lengyel E, Peter ME (2008). The miR-200 family determines the epithelial phenotype of cancer cells by targeting the E-cadherin repressors ZEB1 and ZEB2. Genes Dev..

[9] Ganesan R, Mallets E, Gomez-Cambronero J (2016). The transcription factors Slug (SNAI2) and Snail (SNAI1) regulate phospholipase D (PLD) promoter in opposite ways towards cancer cell invasion. Mol. Oncol..

[10] Wei SC (2015). Matrix stiffness drives epithelial–mesenchymal transition and tumour metastasis through a TWIST1-G3BP2 mechanotransduction pathway. Nat. Cell Biol..

[11] Bhowmick NA (2001). Transforming growth factor-β1 mediates epithelial to mesenchymal transdifferentiation through a RhoA-dependent mechanism. Mol. Biol. Cell.

[12] Lamouille S, Xu J, Derynck R (2014). Molecular mechanisms of epithelial–mesenchymal transition. Nat. Rev. Mol. Cell Biol..

[13] Puisieux A, Brabletz T, Caramel J (2014). Oncogenic roles of EMT-inducing transcription factors. Nat. Cell Biol..

[14] Dongre A, Weinberg RA (2019). New insights into the mechanisms of epithelial–mesenchymal transition and implications for cancer. Nat. Rev. Mol. Cell Biol..

[15] Eriksson JE (2009). Introducing intermediate filaments: from discovery to disease. J. Clin. Investig..

[16] Radice GL (2013). N-cadherin-mediated adhesion and signaling from development to disease: Lessons from mice. Prog. Mol. Biol. Transl. Sci..

[17] Gregory PA (2008). The miR-200 family and miR-205 regulate epithelial to mesenchymal transition by targeting ZEB1 and SIP1. Nat. Cell Biol..

[18] Tam WL, Weinberg RA (2013). The epigenetics of epithelial–mesenchymal plasticity in cancer. Nat. Med..

[19] Serrano-Gomez SJ, Maziveyi M, Alahari SK (2016). Regulation of epithelial–mesenchymal transition through epigenetic and post-translational modifications. Mol. Cancer.

[20] Nieto MA, Huang RY-J, Jackson RA, Thiery JP (2016). EMT: 2016. Cell.

[21] Pastushenko I, Blanpain C (2019). EMT transition states during tumor progression and metastasis. Trends Cell Biol..

[22] Mittal V (2018). Epithelial mesenchymal transition in tumor metastasis. Annu. Rev. Pathol. Mech. Dis..

[23] Li H (2017). Reference component analysis of single-cell transcriptomes elucidates cellular heterogeneity in human colorectal tumors. Nat. Genet..

[24] Calon A (2015). Stromal gene expression defines poor-prognosis subtypes in colorectal cancer. Nat. Genet..

[25] Isella C (2015). Stromal contribution to the colorectal cancer transcriptome. Nat. Genet..

[26] Eischen CM (2016). Genome stability requires p53. Cold Spring Harb. Perspect. Med..

[27] Rooney MS, Shukla SA, Wu CJ, Getz G, Hacohen N (2015). Molecular and genetic properties of tumors associated with local immune cytolytic activity. Cell.

[28] Barretina J (2012). The Cancer Cell Line Encyclopedia enables predictive modelling of anticancer drug sensitivity. Nature.

[29] Li P-D (2018). HOXC6 predicts invasion and poor survival in hepatocellular carcinoma by driving epithelial–mesenchymal transition. Aging.

[30] You X (2020). MicroRNA-495 confers inhibitory effects on cancer stem cells in oral squamous cell carcinoma through the HOXC6-mediated TGF-β signaling pathway. Stem Cell Res. Ther..

[31] Zhang F (2018). HOXC6 gene silencing inhibits epithelial–mesenchymal transition and cell viability through the TGF-β/smad signaling pathway in cervical carcinoma cells. Cancer Cell Int..

[32] Heidary Arash E, Shiban A, Song S, Attisano L (2017). MARK4 inhibits Hippo signaling to promote proliferation and migration of breast cancer cells. EMBO Rep..

[33] Mehrpour M, Codogno P (2010). Prion protein: From physiology to cancer biology. Cancer Lett..

[34] Du L (2013). CD44-Positive cancer stem cells expressing cellular prion protein contribute to metastatic capacity in colorectal cancer. Cancer Res..

[35] Hay ED (1995). An overview of epithelio-mesenchymal transformation. Acta Anat. (Basel).

[36] Mani SA (2008). The epithelial–mesenchymal transition generates cells with properties of stem cells. Cell.

[37] Nelson CE (1996). Analysis of Hox gene expression in the chick limb bud. Development.

[38] Naz F, Anjum F, Islam A, Ahmad F, Hassan MdI (2013). Microtubule affinity-regulating kinase 4: Structure, function, and regulation. Cell Biochem. Biophys..

[39] Dumont NA (2015). Dystrophin expression in muscle stem cells regulates their polarity and asymmetric division. Nat. Med..

[40] Turner N, Grose R (2010). Fibroblast growth factor signalling: from development to cancer. Nat. Rev. Cancer.

[41] Suo D (2020). HOXC10 upregulation confers resistance to chemoradiotherapy in ESCC tumor cells and predicts poor prognosis. Oncogene.

[42] Thiery JP (2002). Epithelial–mesenchymal transitions in tumour progression. Nat. Rev. Cancer.

[43] Ashley J (2018). Retrovirus-like gag protein Arc1 binds RNA and traffics across synaptic boutons. Cell.

[44] Wall MJ, Corrêa SAL (2018). The mechanistic link between Arc/Arg3.1 expression and AMPA receptor endocytosis. Semin. Cell Dev. Biol..

[45] Pastuzyn ED (2018). The neuronal gene Arc encodes a repurposed retrotransposon gag protein that mediates intercellular RNA transfer. Cell.

[46] Nishida N (2012). Microarray analysis of colorectal cancer stromal tissue reveals upregulation of two oncogenic miRNA clusters. Clin. Cancer Res..

[47] Gentles AJ (2015). The prognostic landscape of genes and infiltrating immune cells across human cancers. Nat. Med..

[48] Miyoshi H, Blömer U, Takahashi M, Gage FH, Verma IM (1998). Development of a self-inactivating lentivirus vector. J. Virol..

[49] Hashiguchi Y (2020). Japanese Society for Cancer of the Colon and Rectum (JSCCR) guidelines 2019 for the treatment of colorectal cancer. Int. J. Clin. Oncol..

[50] Li B, Dewey CN (2011). RSEM: accurate transcript quantification from RNA-Seq data with or without a reference genome. BMC Bioinform..

