# Supplementary material for: Comprehensive profiling of novel epithelial–mesenchymal transition mediators and their clinical significance in colorectal cancer
Source: Sci Rep. 2021 Jun 3;11:11759. doi: 10.1038/s41598-021-91102-9 (PMC8175715; doi:10.1038/s41598-021-91102-9)
Supplement: Supplementary file 1 — Supplementary Information. [file 41598_2021_91102_MOESM1_ESM.pdf]

Supplementary information

Comprehensive Profiling of Novel Epithelial-Mesenchymal Transition Mediators and Their Clinical Significance in Colorectal Cancer

Satoshi Ishikawa, Naohiro Nishida, Shiki Fujino, Takayuki Ogino, Hidekazu Takahashi, Norikatsu Miyoshi, Mamoru Uemura, Taroh Satoh, Hirofumi Yamamoto, Tsunekazu Mizushima, Yuichiro Doki, Hidetoshi Eguchi

Table of Contents:

Supplementary Figures

Supplementary Figure 1-11

Supplementary Table

Supplementary Table 1

Supplementary Text

Supplementary Text 1, 2

Supplementary References

Supplementary Figures

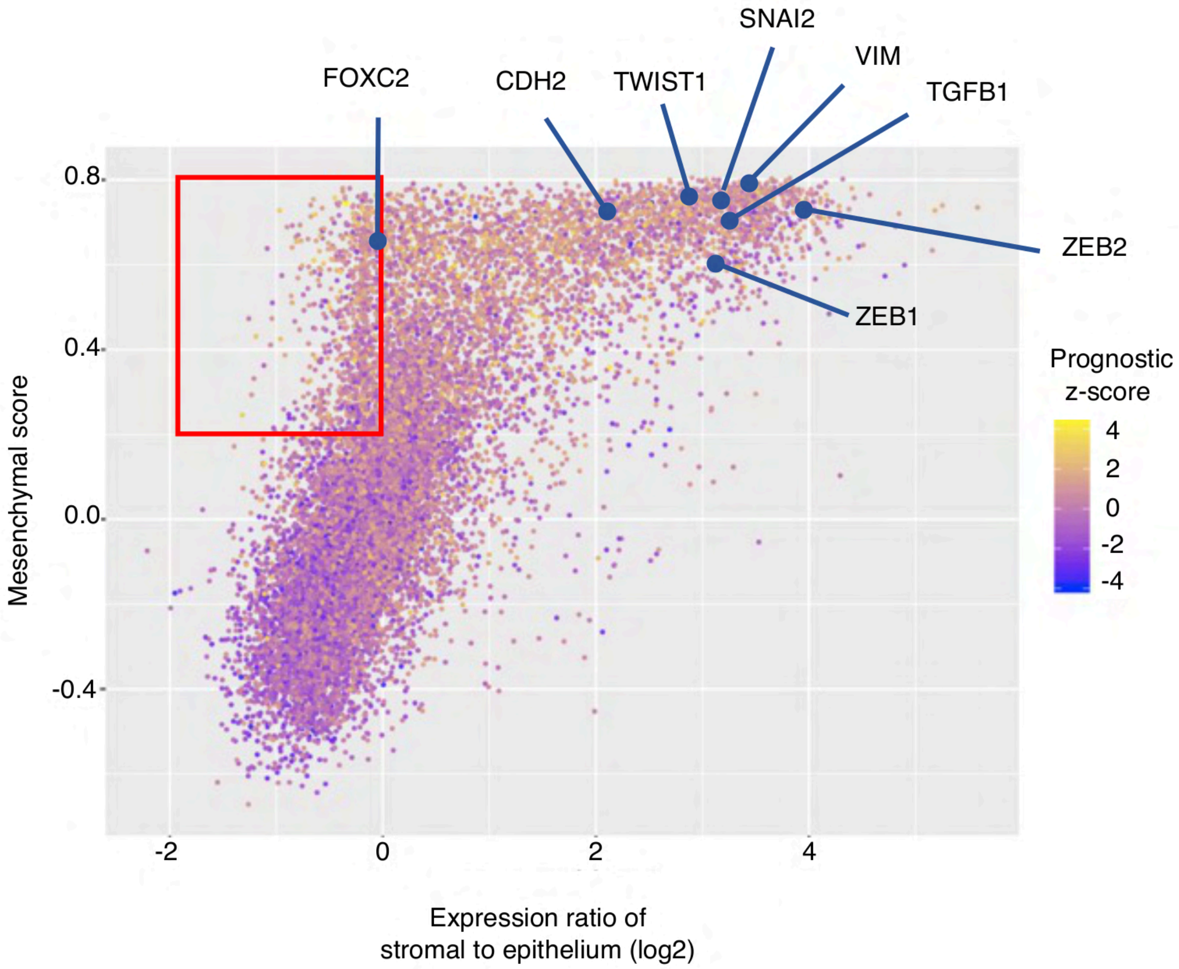

Supplementary Fig. 1: The location of known mesenchymal genes in Figure 1d. The horizontal axis represents log fold change in the expression of all genes of the cancer stroma to epithelium, and the vertical axis represents mesenchymal scores. Colors of points indicate z-scores of prognosis. The red frame includes genes with mesenchymal scores > 0.3, the expression ratio in stroma to epithelium (log2) < 0, and z-scores of prognosis > 1.96. Known mesenchymal markers: VIM, SNAI2, ZEB1, ZEB2, TWIST1, CDH2, TGFB1, and FOXC2.

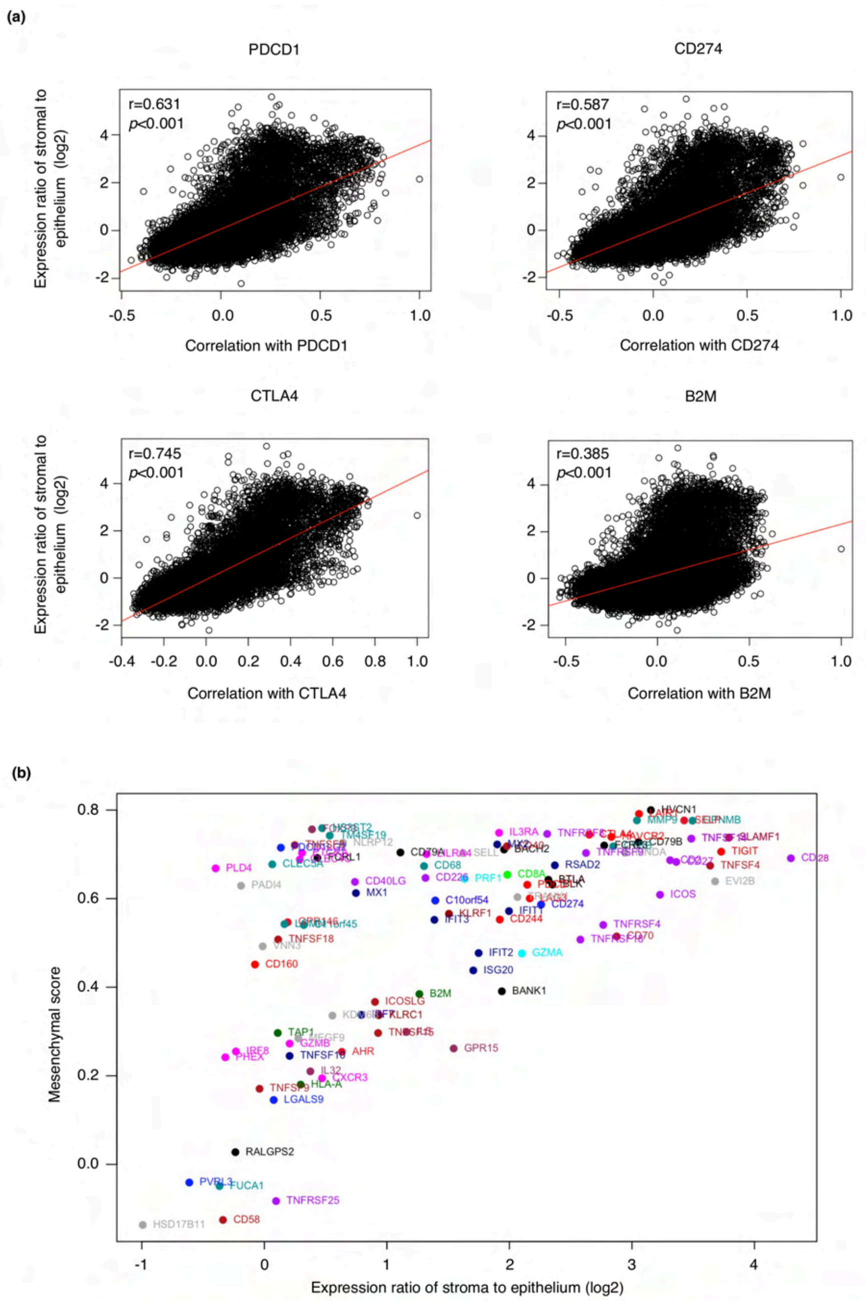

B cells	CD4+ Regulatory T cells	CD8+ T cells	Macrophages	Neutrophils	NK cells	pDCs	MHC Class I
CD79B	FOXP3	CD8A	FUCA1	KDM6B	KLRF1	LILRA4	HLA-A
BTLA	C15orf53		MMP9	HSD17B11	KLRC1	CLEC4C	B2M
FCRL3	IL5		LGMN	EVI2B		PLD4	TAP1
BANK1	CTLA4		HS3ST2	MNDA		PHEX	
CD79A	IL32		TM4SF19	MEGF9		IL3RA	
BLK	GPR15		CLEC5A	SELL		PTCRA	
RALGPS2	IL4		GPNMB	NLRP12		IRF8	
FCRL1			C11orf45	PADI4		IRF7	
HVCN1			CD68	TRANK1		GZMB	
BACH2			CYBB	VNN3		CXCR3	

Co-stimulation, APC	Co-stimulation, T cell	Co-inhibition, APC	Co-inhibition, T cell	Type I IFN Response	Type II IFN Response	Cytolytic Activity
ICOSLG	ICOS	PDCD1LG2	LAG3	MX1	GPR146	GZMA
CD70	CD28	CD274	CTLA4	TNFSF10	SELP	PRF1
TNFSF14	CD27	C10orf54	CD274	RSAD2	AHR	
CD40	TNFSF14	LGALS9	CD160	IFIT1		
TNFSF9	CD40LG	PVRL3	BTLA	IFIT3		
TNFSF4	TNFRSF9		C10orf54	IFIT2		
TNFSF15	TNFRSF4		LAIR1	IRF7		
TNFSF18	TNFRSF25		HAVCR2	DDX4		
TNFSF8	TNFRSF18		CD244	MX2		
SLAMF1	TNFRSF8		TIGIT	ISG20		
CD58	SLAMF1		PDCD1			
	CD2					
	CD226					

Supplementary Fig. 2: Mesenchymal scores in genes associated with immune response. (a) Mesenchymal scores in four representative genes associated with immune response. The horizontal axis represents Pearson's correlation coefficients between four target genes and all other genes in CRC in TCGA dataset, and the vertical axis represents log fold change in all genes' expression in cancer stroma to epithelium in GSE35602. The targets are genes associated with immune response (PDCD1, CD274, CTLA4, and B2M). Correlation coefficients, "Mesenchymal scores," were calculated as r = 0.631, $p < 0.001$ for PDCD1, and r = 0.587, $p < 0.001$ for CD274, r = 0.745, $p < 0.001$ for CTLA4, and r = 0.385, $p < 0.001$ for B2M. (b) Immune related genes are plotted as Figure 1d. Genes are color-coded according to their functions. Horizontal axis, stromal enrichment (expression ratio of stroma to epithelium); Vertical axis, mesenchymal score (Upper). The function of each gene is listed in lower panel.

CRC: colorectal cancer; TCGA: The Cancer Genome Atlas; NK: natural killer; pDCs: plasmacytoid dendritic cells; MHC: major histocompatibility complex; APC: Antigen presenting cell; IFN: interferon

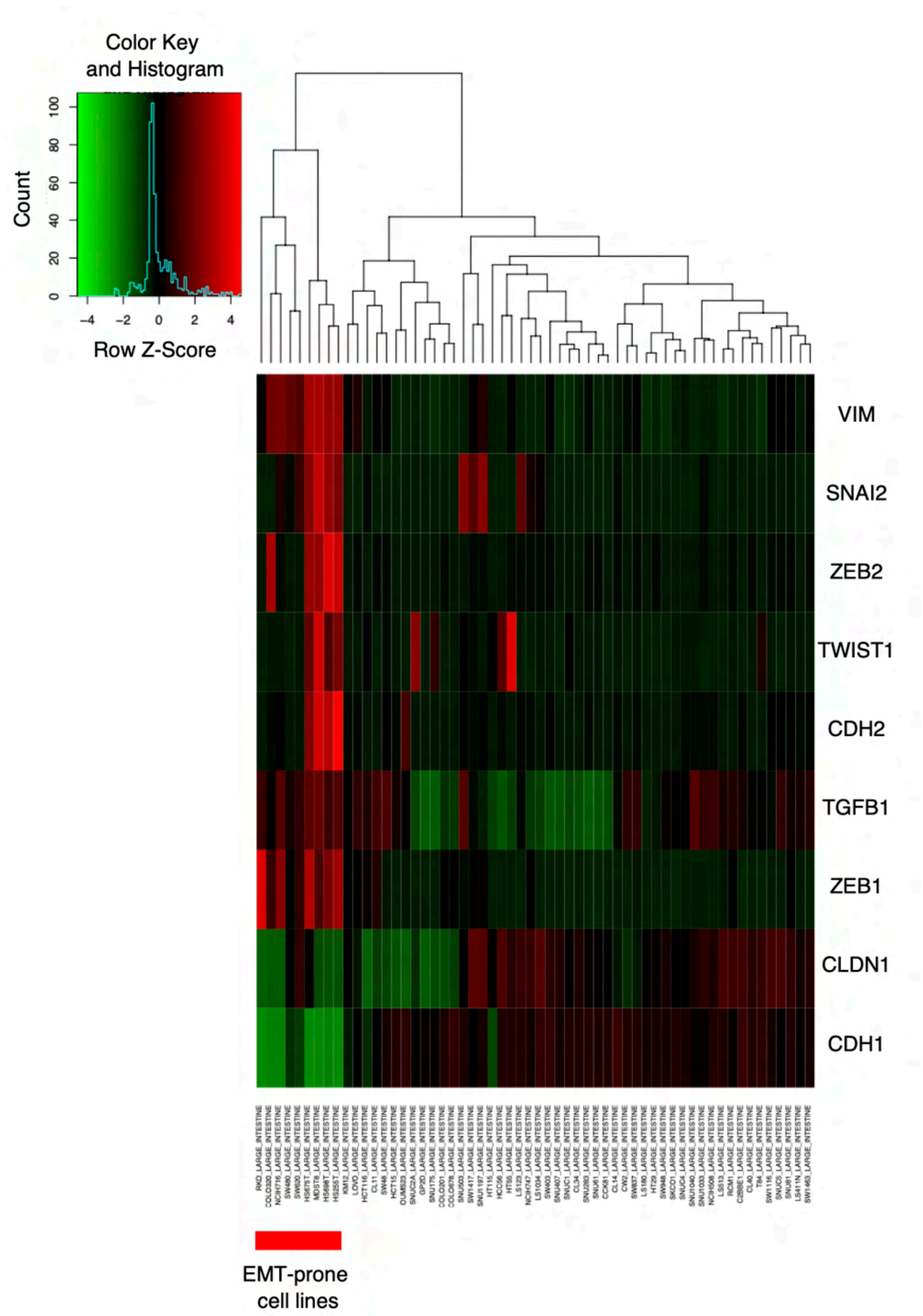

Supplementary Fig. 3: Definition of EMT-prone cell lines. We defined EMT-prone cell lines by unsupervised hierarchical clustering using expression data of known EMT-related genes in CRC cell lines from CCLE dataset. EMT: epithelial-mesenchymal transition; CRC: colorectal cancer; CCLE: Cancer Cell Line Encyclopedia

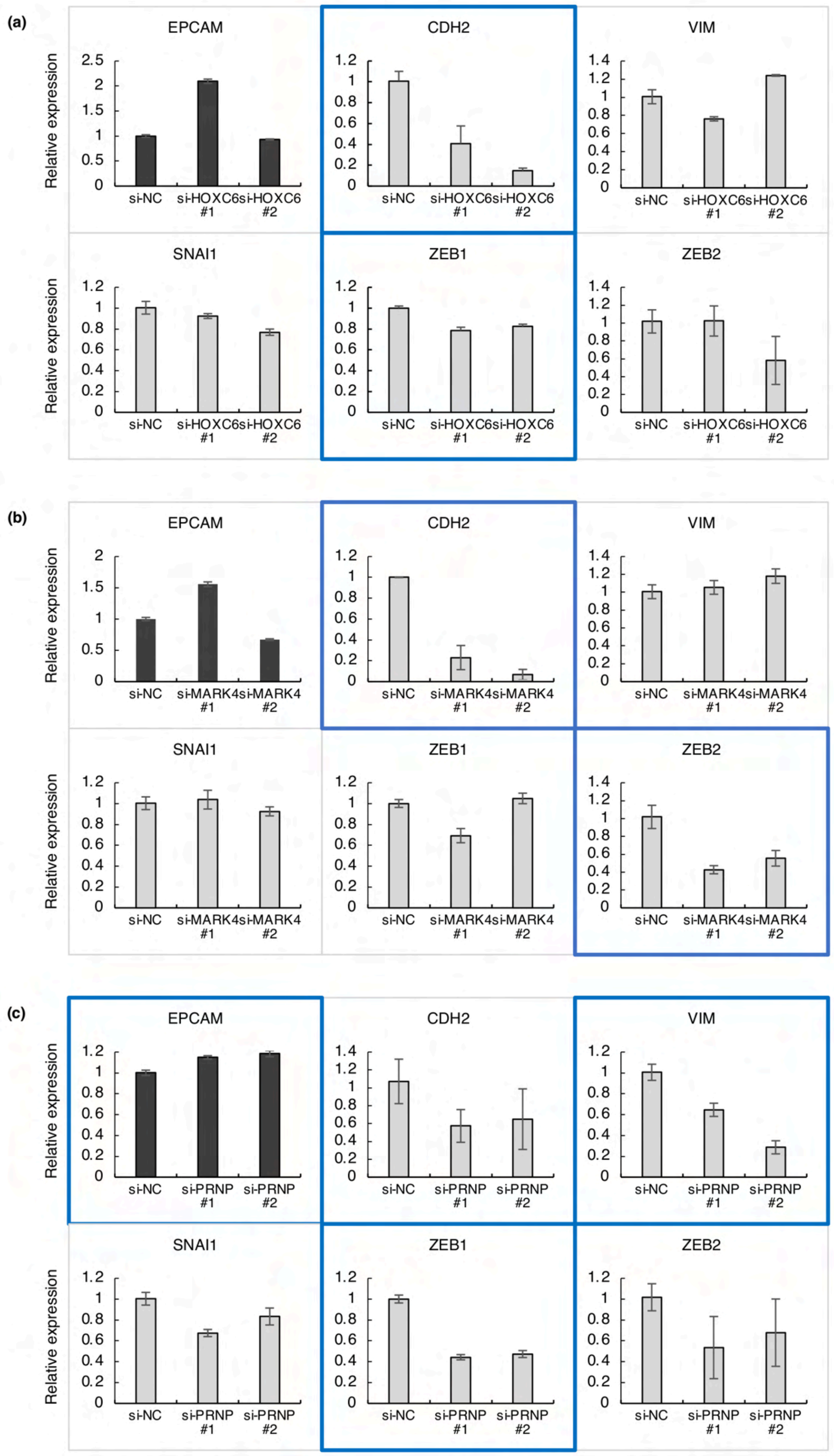
(a)
EPCAM
CDH2
VIM
SNAI1
ZEB1
ZEB2
Relative expression
si-NC
si-HOXC6 #1
si-HOXC6 #2
(b)
EPCAM
CDH2
VIM
SNAI1
ZEB1
ZEB2
Relative expression
si-NC
si-MARK4 #1
si-MARK4 #2
(c)
EPCAM
CDH2
VIM
SNAI1
ZEB1
ZEB2
Relative expression
si-NC
si-PRNP #1
si-PRNP #2

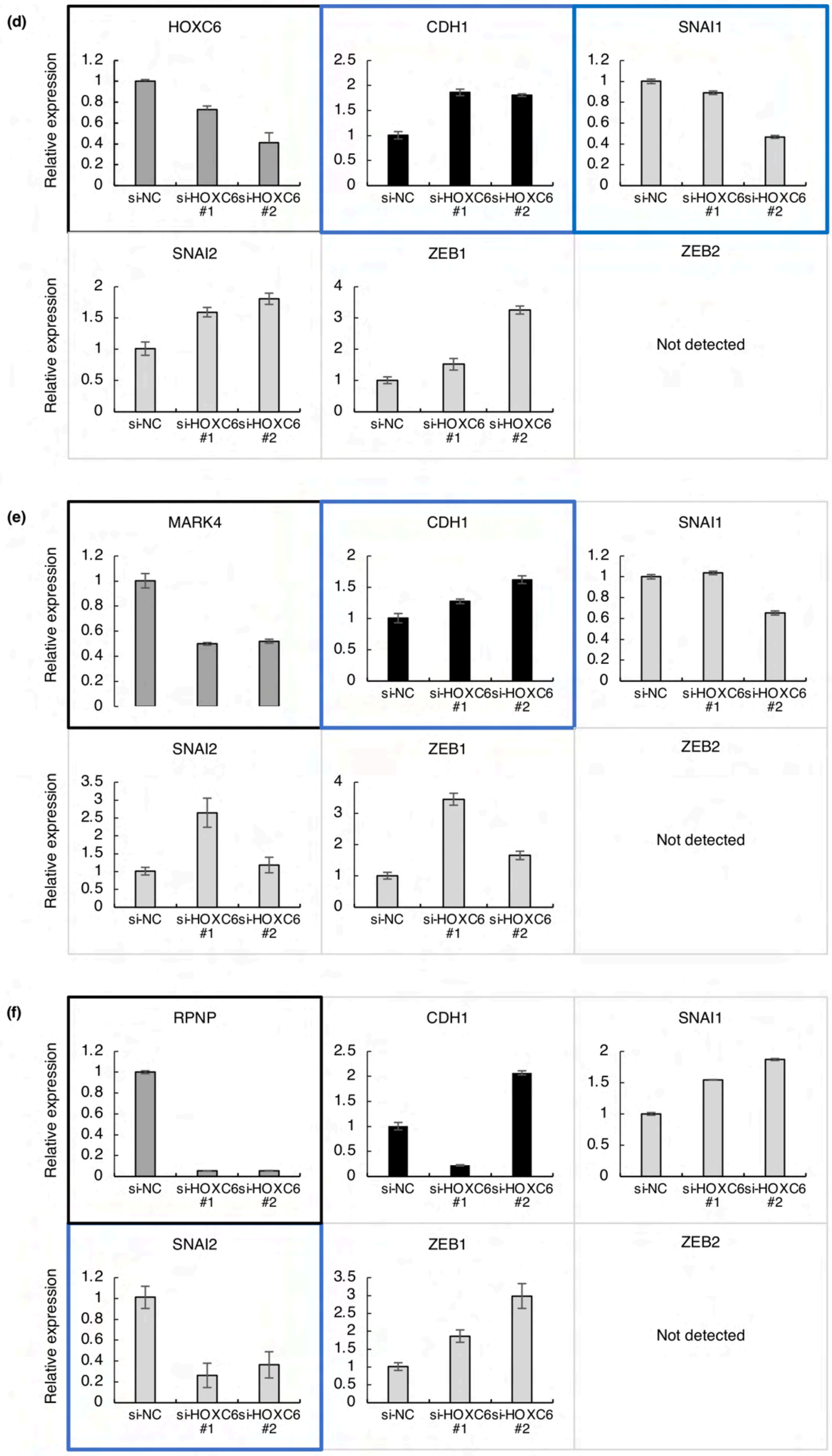
(d)
HOXC6
CDH1
SNAI1
SNAI2
ZEB1
ZEB2
Not detected
Relative expression
si-NC
si-HOXC6 #1
si-HOXC6 #2
(e)
MARK4
CDH1
SNAI1
SNAI2
ZEB1
ZEB2
Not detected
Relative expression
si-NC
si-HOXC6 #1
si-HOXC6 #2
(f)
RPNP
CDH1
SNAI1
SNAI2
ZEB1
ZEB2
Not detected
Relative expression
si-NC
si-HOXC6 #1
si-HOXC6 #2

Supplementary Fig. 4: (a, b, c) Alteration in expression of another epithelial marker (EPCAM) and mesenchymal markers (CDH2, VIM, SNAI1, ZEB1, and ZEB2) by knockdown of (a) HOXC6, (b) MARK4, and (c) PRNP in HCT116. (b, c, d) Alteration in expression of CDH1 and mesenchymal markers (SNAI1, SNAI2, and ZEB1) by knockdown of (a) HOXC6, (b) MARK4, and (c) PRNP in DLD1.

experiments were conducted in triplicate. Error bars show standard errors of the mean. Blue boxes show repressed mesenchymal markers or enhanced epithelial markers by gene-knockdown experiments.

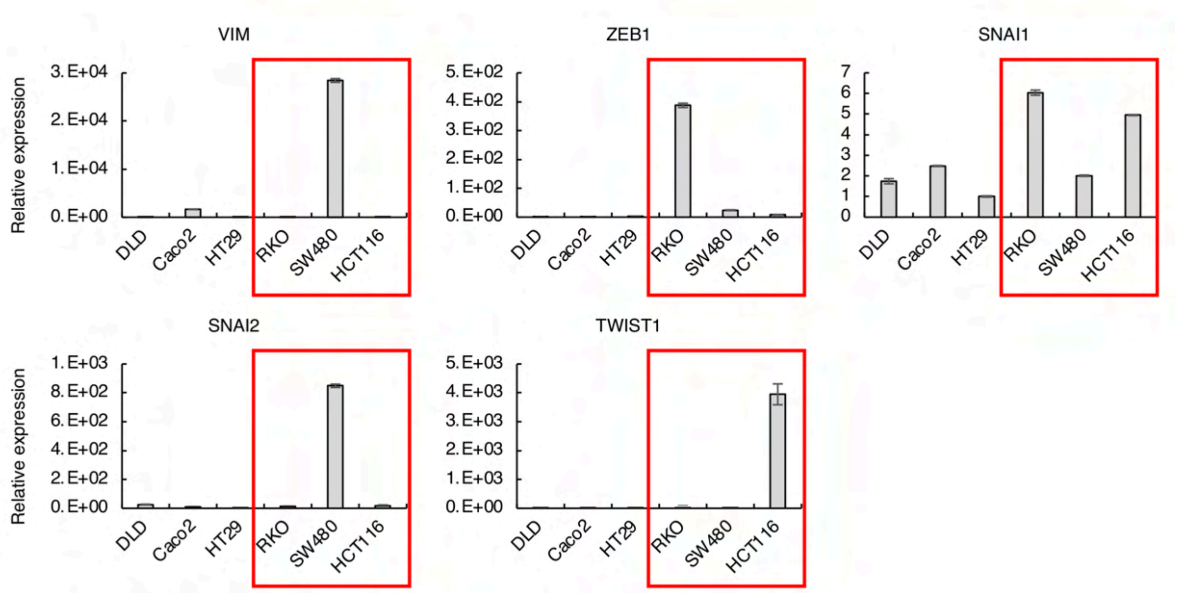

Supplementary Fig. 5: Expression of mesenchymal markers in six CRC cell lines (DLD-1, Caco2, HT29, RKO, SW480, and HCT116). Red boxes indicate cell lines with relatively high expression of ARC.
Experiments were conducted in triplicate. Error bars show standard errors of the mean.

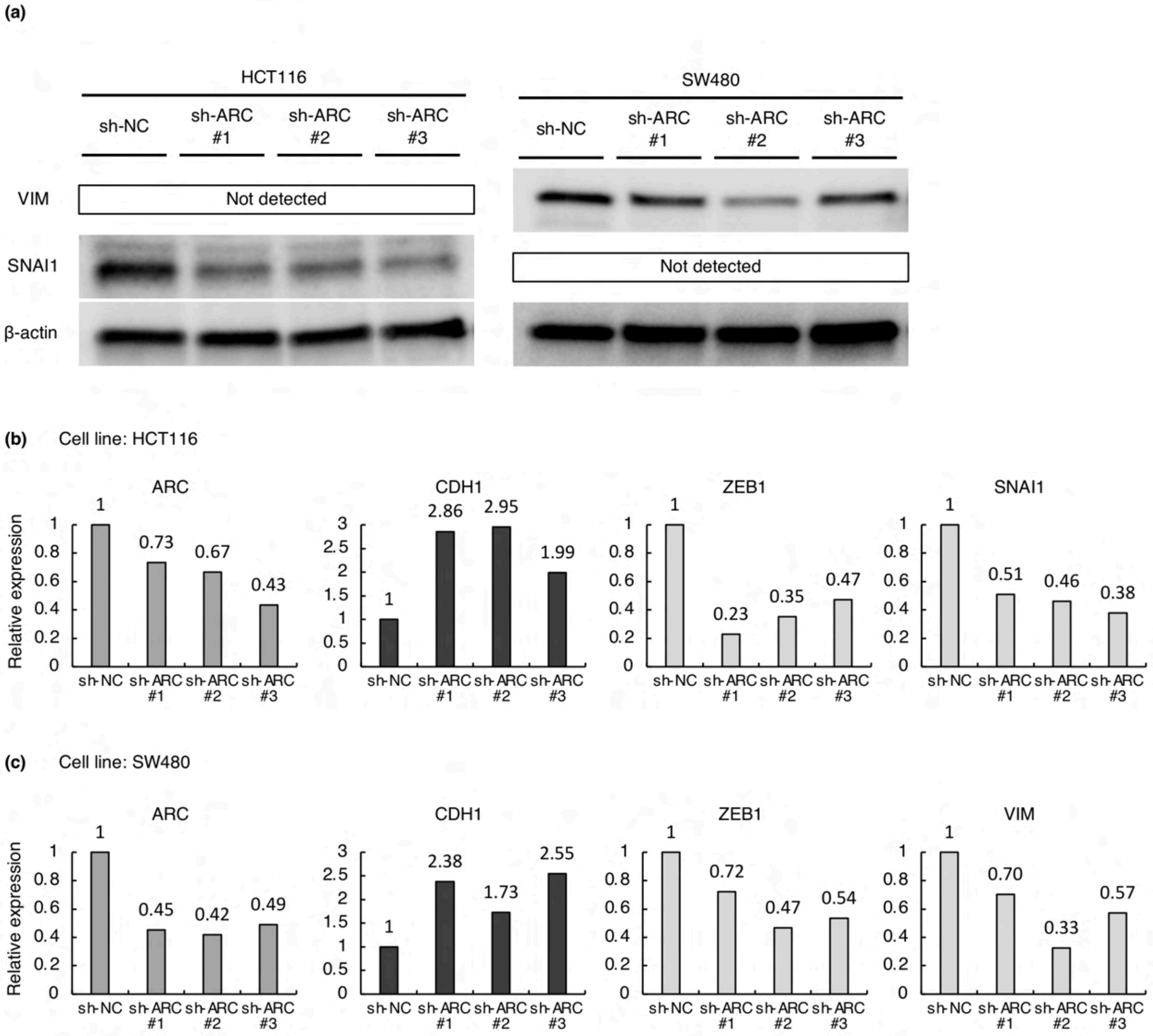

Supplementary Fig. 6: (a) Alteration in expression of mesenchymal markers by knockdown of ARC using shRNAs in western blot analysis. Full-length blots are presented in Supplementary Fig. 11a. (b, c) Quantification of western blot analysis in Fig. 3b and Supplementary Fig. 6a.

Sh: short hairpin; NC: negative control

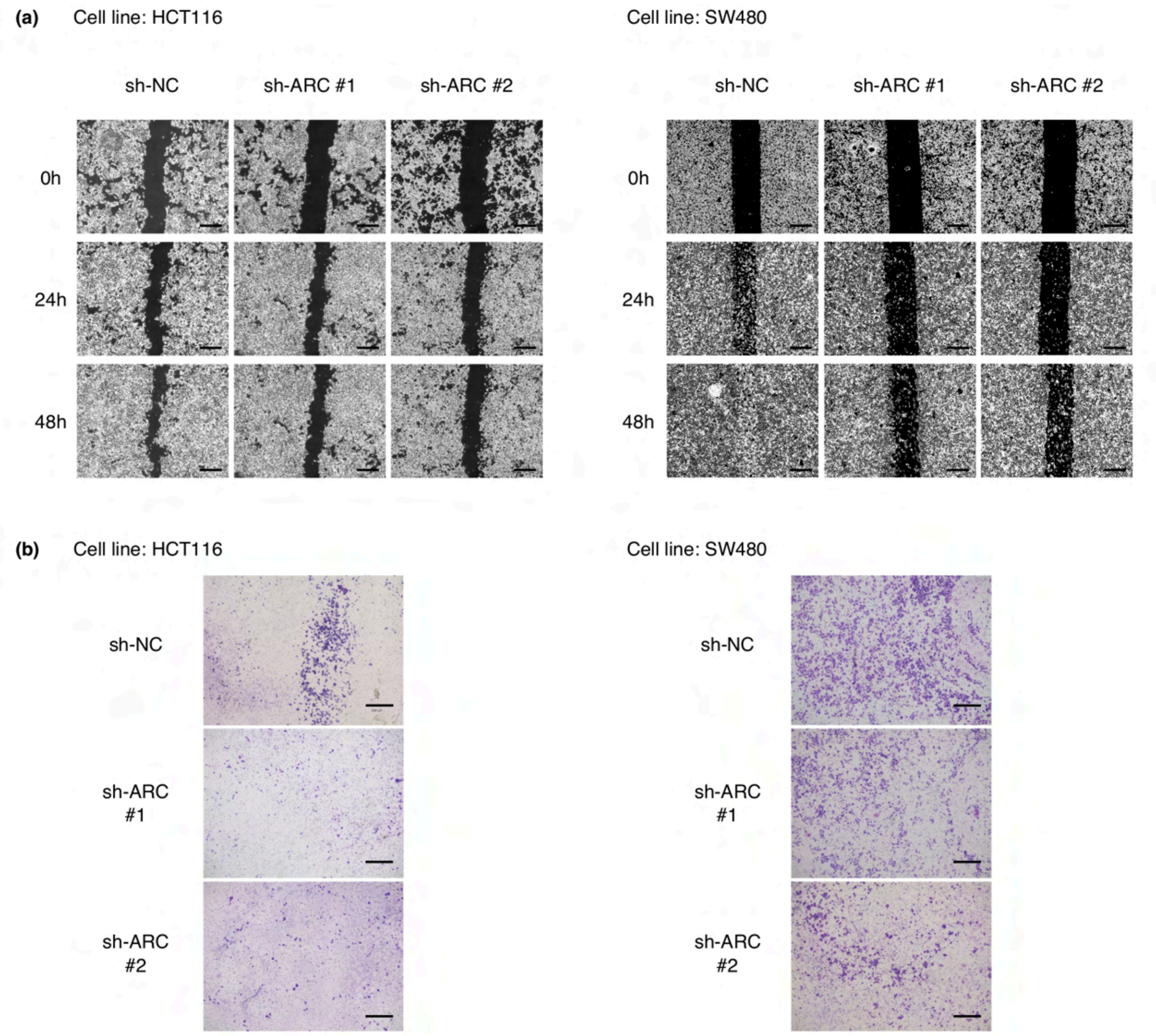

Supplementary Fig. 7: (a) Scratch wound healing assay in HCT116 and SW480 cells expressing sh-NC and sh-ARC. Wound healing width is shown in Fig. 4c. (b) Cell invasion assay in HCT116 and SW480 cells expressing sh-NC and sh-ARC. Counts of invasion cells per field are shown in Fig. 4d.

All experiments were conducted in triplicate. Scale bars indicate 500 μm.

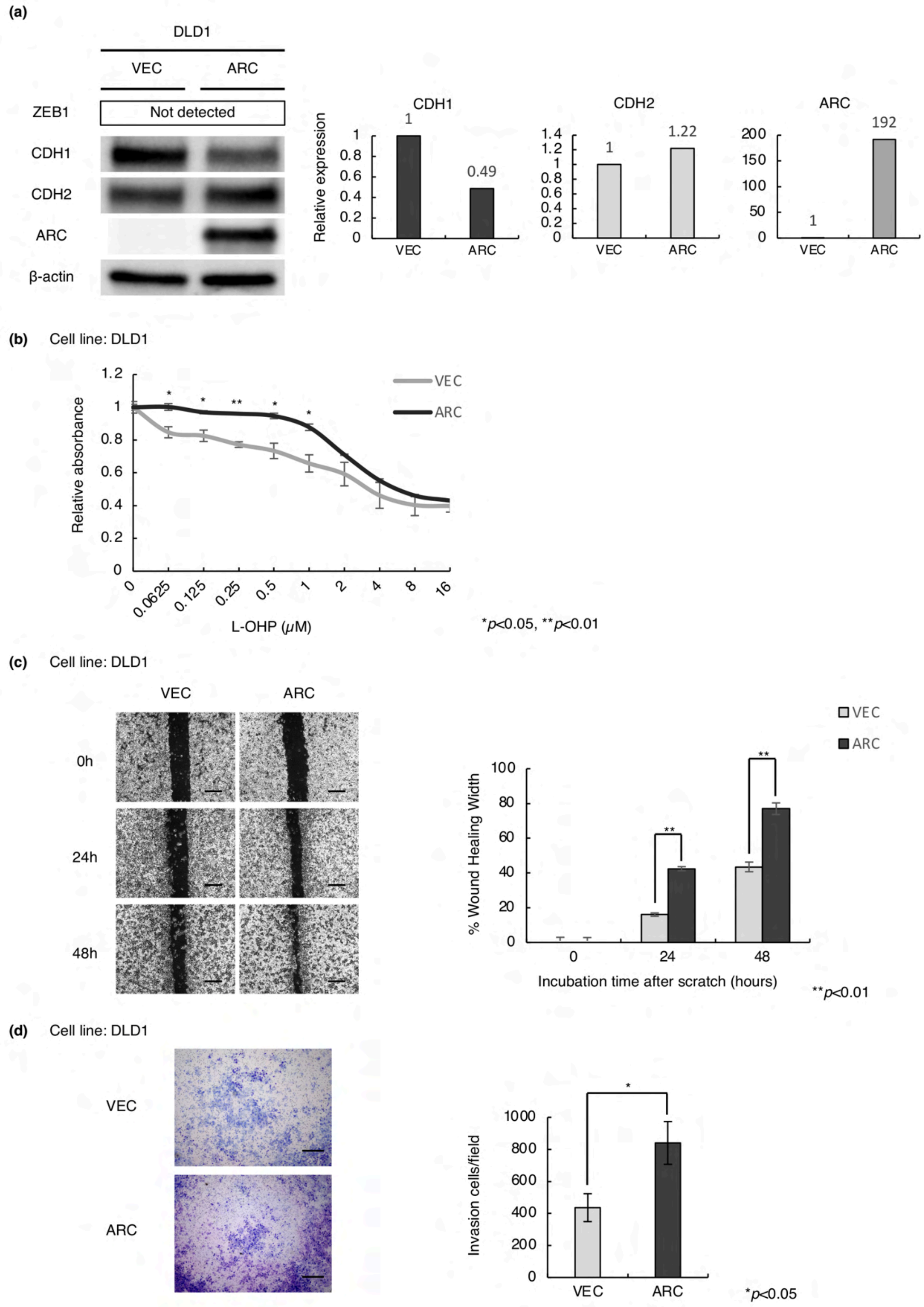

Supplementary Fig. 8: Alteration of cell function by ARC-overexpression in DLD1 cells.

(a) Alteration in CDH1 and CDH2 expression by overexpression of ARC in western blot

analysis. Full-length blots are presented in Supplementary Fig. 11c. (b) Chemosensitivity assay to L-OHP in ARC-overexpressed cells. (c) Scratch wound healing assay in ARC-overexpressed cells. Scale bars indicate 500 μm. (d) Cell invasion assay in ARC-overexpressed cells. Scale bars indicate 500 μm.

All experiments were conducted in triplicate. Error bars represent standard errors of the mean. Asterisks denote significant differences using the unpaired 2-tail t-test (*$p < 0.05$, **$p < 0.01$). VEC: empty vector; L-OHP: oxaliplatin

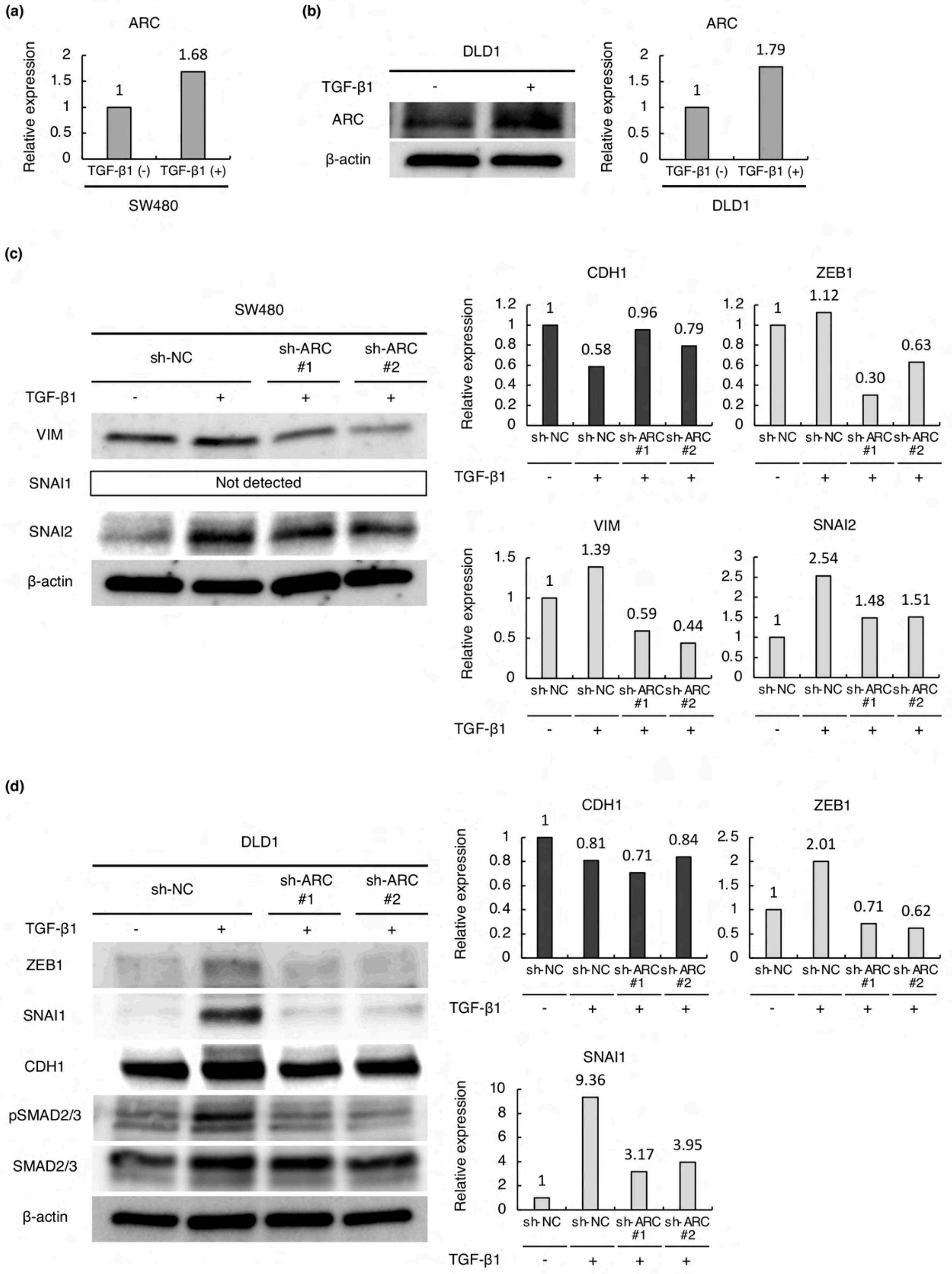
(a)
ARC
Relative expression
1
1.68
TGF-β1 (-) TGF-β1 (+)
SW480
(b)
DLD1
TGF-β1 - +
ARC
β-actin
ARC
Relative expression
1
1.79
TGF-β1 (-) TGF-β1 (+)
DLD1
(c)
SW480
sh-NC
sh-ARC #1
sh-ARC #2
TGF-β1 - + + +
VIM
SNAI1
Not detected
SNAI2
β-actin
CDH1
1 0.58 0.96 0.79
ZEB1
1 1.12 0.30 0.63
VIM
1 1.39 0.59 0.44
SNAI2
1 2.54 1.48 1.51
sh-NC sh-NC sh-ARC #1 sh-ARC #2
TGF-β1 - + + +
(d)
DLD1
sh-NC
sh-ARC #1
sh-ARC #2
TGF-β1 - + + +
ZEB1
SNAI1
CDH1
pSMAD2/3
SMAD2/3
β-actin
CDH1
1 0.81 0.71 0.84
ZEB1
1 2.01 0.71 0.62
SNAI1
1 9.36 3.17 3.95
sh-NC sh-NC sh-ARC #1 sh-ARC #2
TGF-β1 - + + +

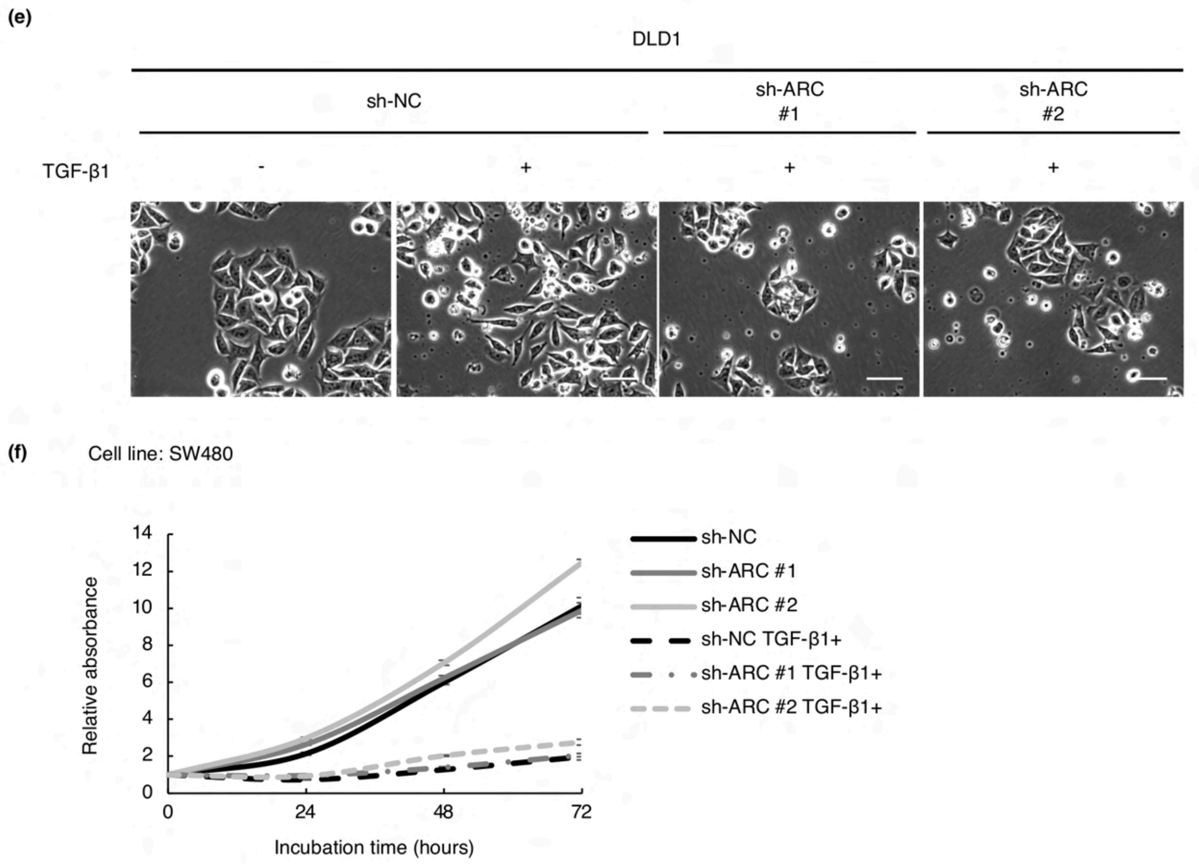

Supplementary Fig. 9: Involvement of ARC in TGF-β-mediated EMT pathway in SW480 and DLD1. (a) Quantification of western blot analysis in Figure 5b. (b) Alteration of ARC expression in DLD1 by TGF-β1 in western blot analysis. Full-length blots are presented in Supplementary Fig. 11e. (c) Alteration of other mesenchymal markers in addition to Figure 5c and quantification of western blot analysis. (d) Alteration of ZEB1 and SNAI1 expression in sh-NC and sh-ARC DLD1 cells in response to TGF-β1 in western blot analysis. (e) TGF-β1-induced change in cell shape in sh-NC and sh-ARC SW480 cell lines. (f) Inhibition of cell proliferation by TGF-β1.

Experiments were conducted in triplicate. TGF: transforming growth factor; EMT: epithelial-mesenchymal transition; sh: short hairpin; NC: negative control

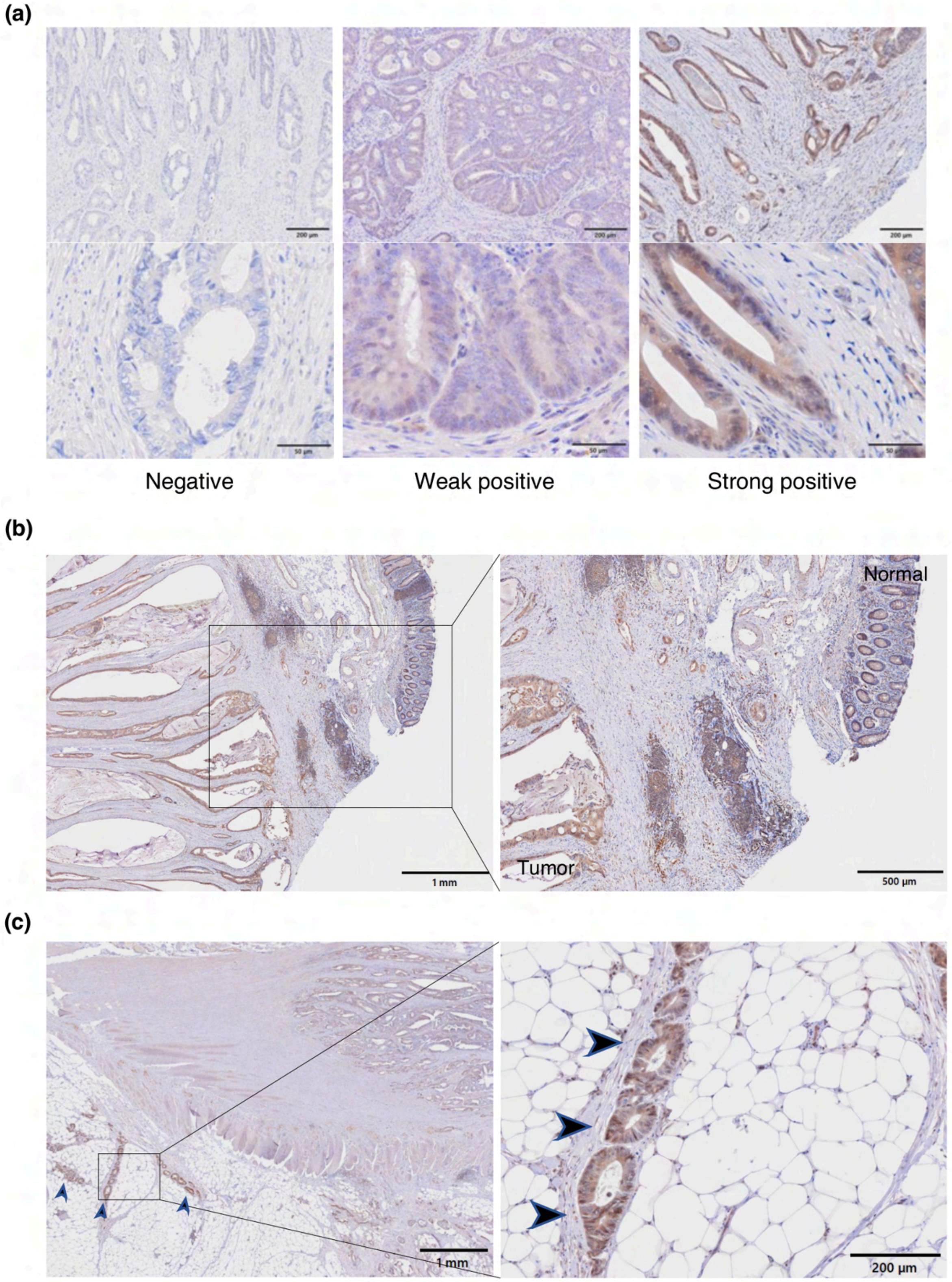
(a)
Negative
Weak positive
Strong positive
(b)
Normal
Tumor
1 mm
500 µm
(c)
1 mm
200 µm

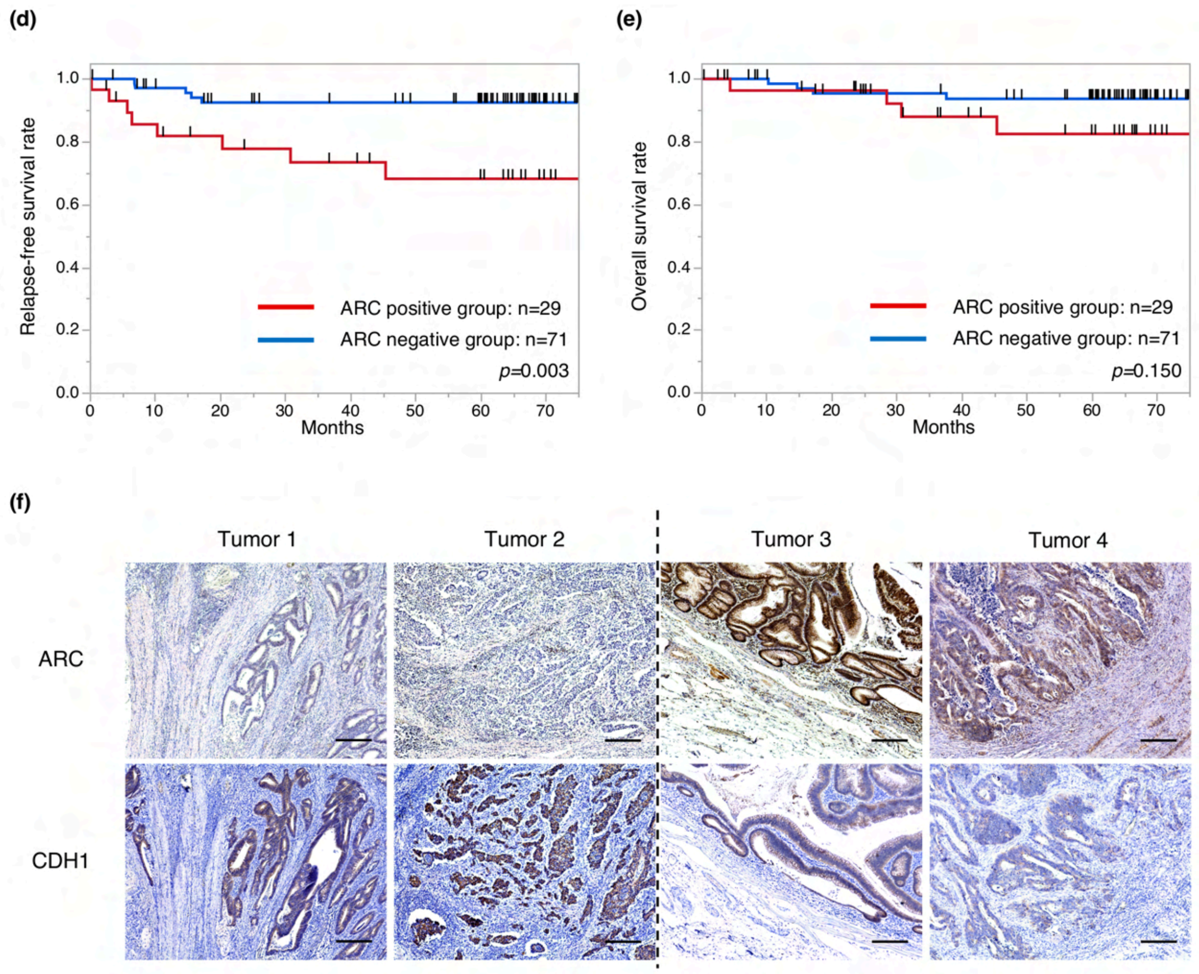

Supplementary Fig. 10: Association between ARC expression in IHC and outcomes in CRC patients. (a) The intensity of ARC expression in IHC. We assigned the specimen with the same intensity of staining as the positive control to strong positive group, the unstained specimen to negative group, and the specimen stained weaker than the positive control to weak positive group. The positive control: human brain tissue; Scale bars indicate 200 µm (above) and 50 µm (below). (b) IHC of ARC in tumor and normal tissue in a specimen of CRC. Scale bars indicate 1 mm (left) and 500 µm (right). (c) IHC of ARC in the deepest parts of tumor in a specimen of CRC. Scale bars indicate 1 mm (left) and 200 µm (right). (d) Kaplan-Meier curves for RFS in CRC patients according to ARC expression (p = 0.003). Patients are divided into two groups by the intensity of ARC expression in IHC (negative expression: negative group, weak and strong positive expression: positive group). (e) Kaplan-Meier curves for OS in CRC patients according to ARC expression (p = 0.150). Patients are

divided into two groups by the intensity of ARC expression in IHC. (f) Representative images of IHC for ARC and CDH1. Scale bars indicate 200 µm.

IHC: immunohistochemical staining; RFS: relapse-free survival; OS: overall survival

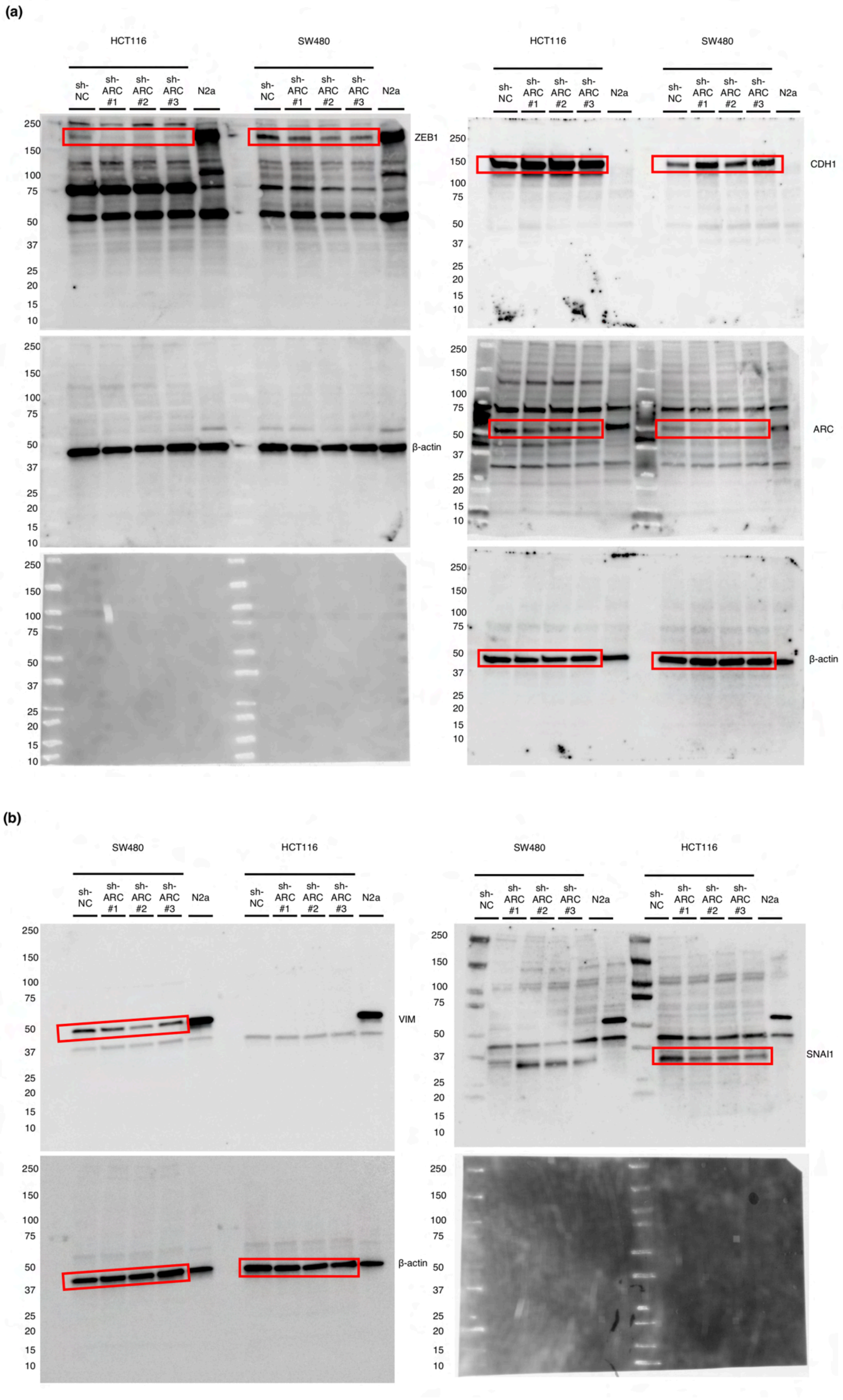
(a)
HCT116
SW480
sh-NC
sh-ARC #1
sh-ARC #2
sh-ARC #3
N2a
ZEB1
CDH1
β-actin
ARC
β-actin
(b)
SW480
HCT116
VIM
SNAI1
β-actin
250
150
100
75
50
37
25
20
15
10

(c)

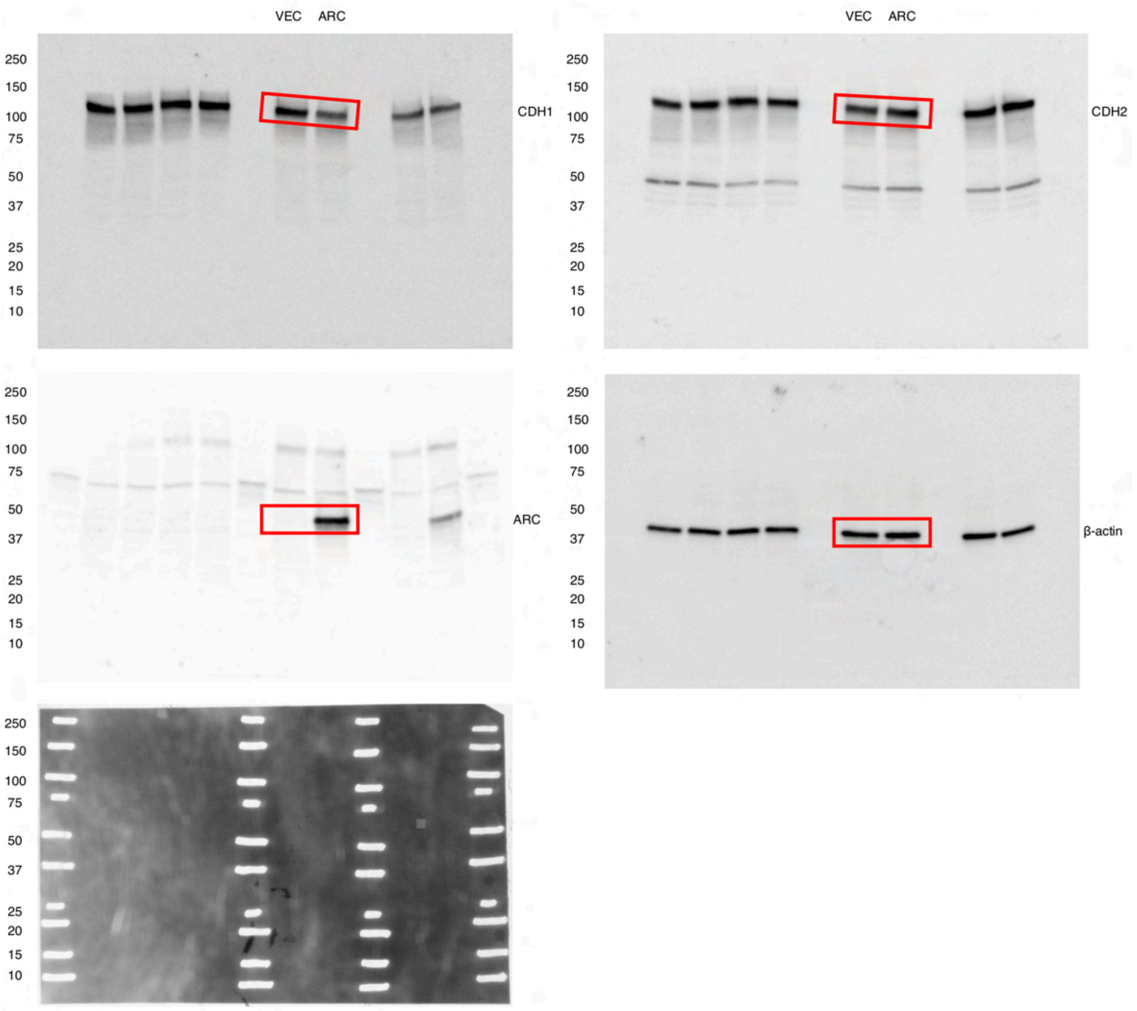
VEC ARC
VEC ARC
250
150
100
75
50
37
25
20
15
10
CDH1
CDH2
ARC
β-actin

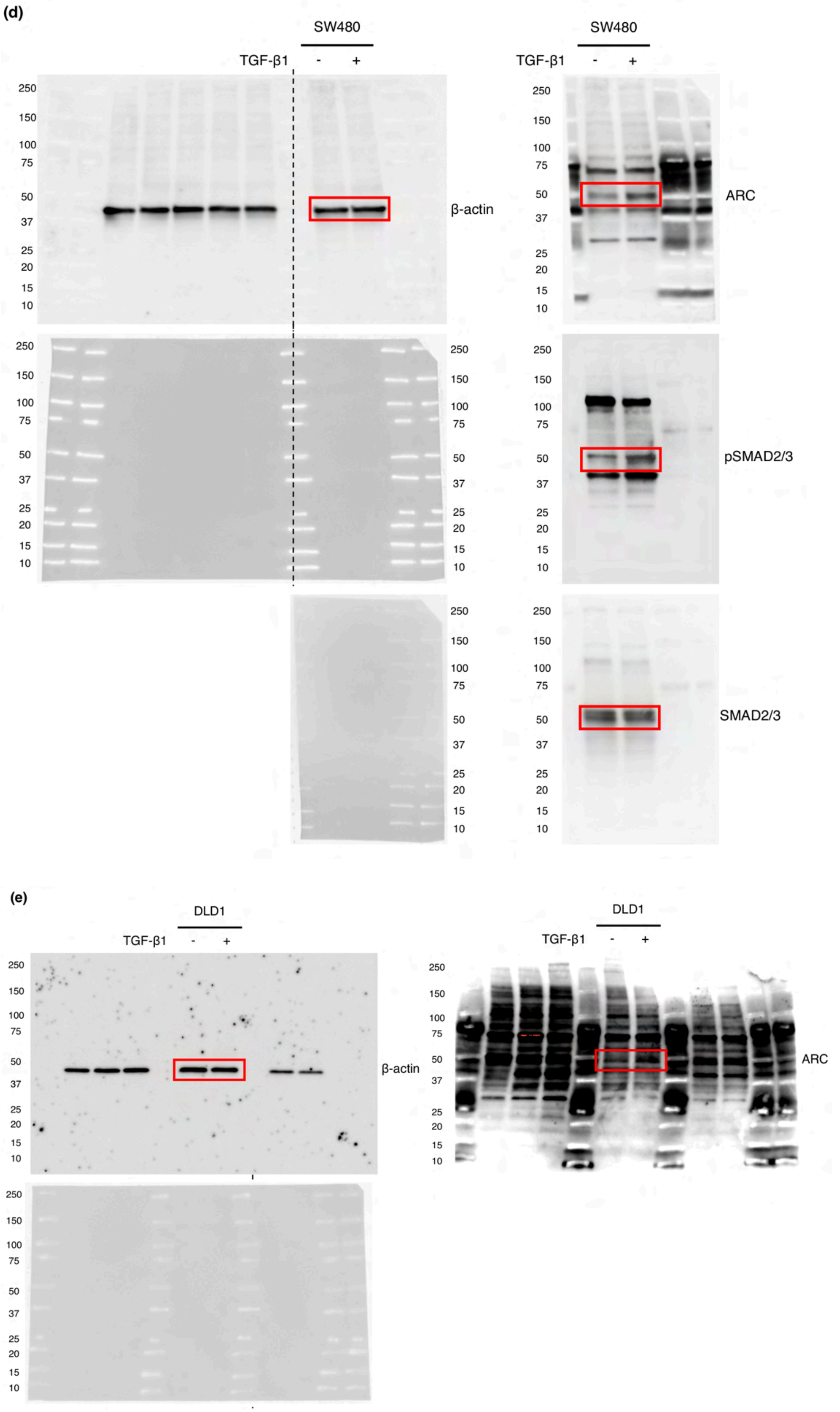
(d)
SW480
TGF-β1 - +
β-actin
SW480
TGF-β1 - +
ARC
pSMAD2/3
SMAD2/3
250
150
100
75
50
37
25
20
15
10
(e)
DLD1
TGF-β1 - +
β-actin
DLD1
TGF-β1 - +
ARC

(f)

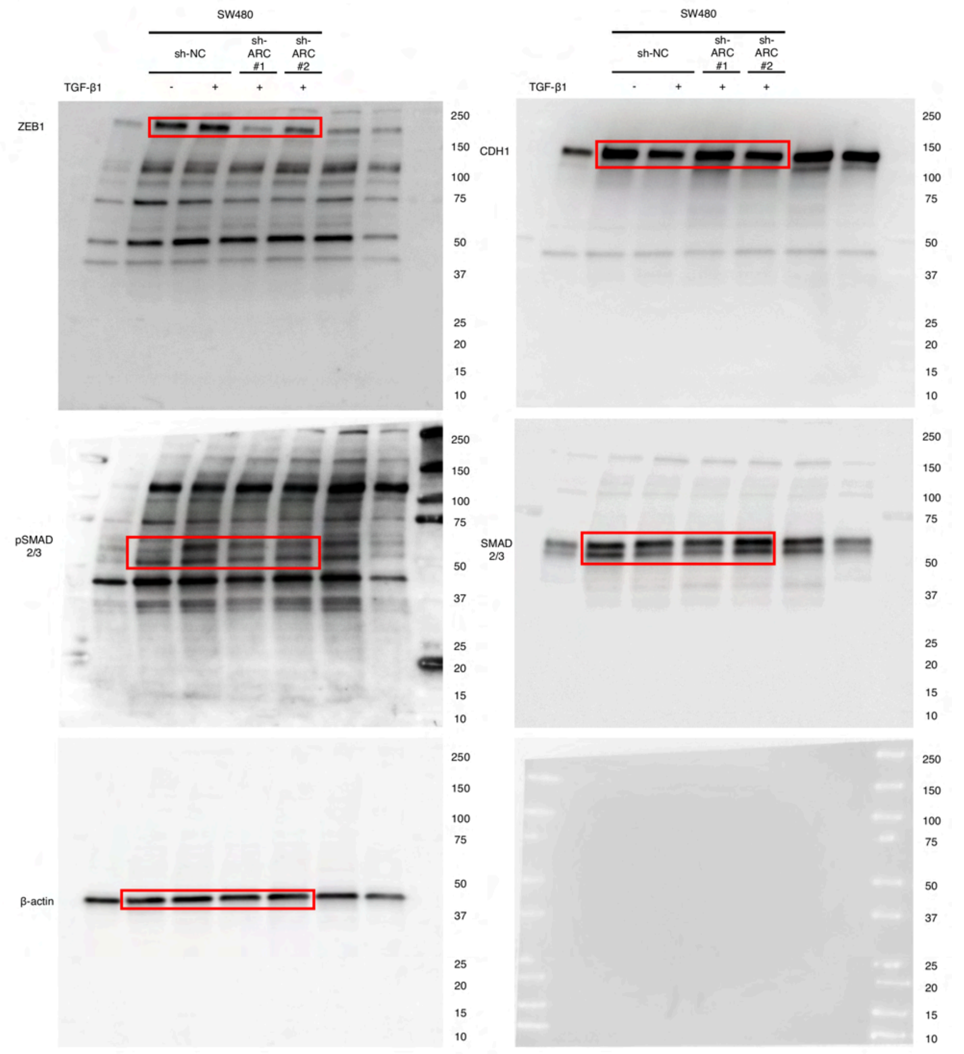
SW480
sh-NC
sh-ARC #1
sh-ARC #2
TGF-β1
- + + +
ZEB1
CDH1
pSMAD 2/3
SMAD 2/3
β-actin
250
150
100
75
50
37
25
20
15
10

(g)

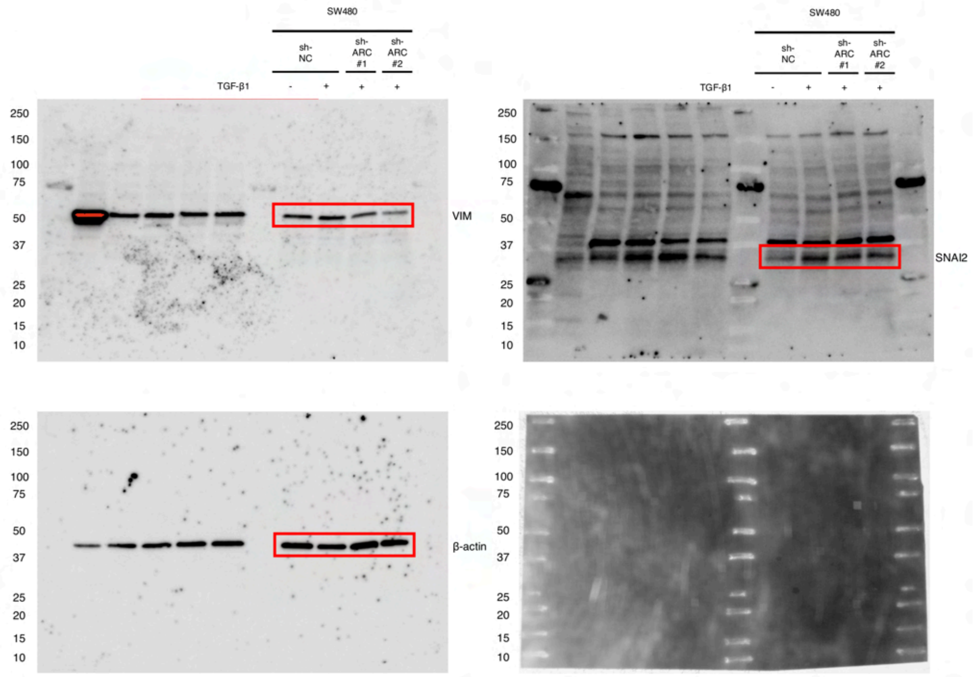
SW480
sh-NC
sh-ARC #1
sh-ARC #2
TGF-β1
- + + +
VIM
SNAI2
β-actin
250
150
100
75
50
37
25
20
15
10

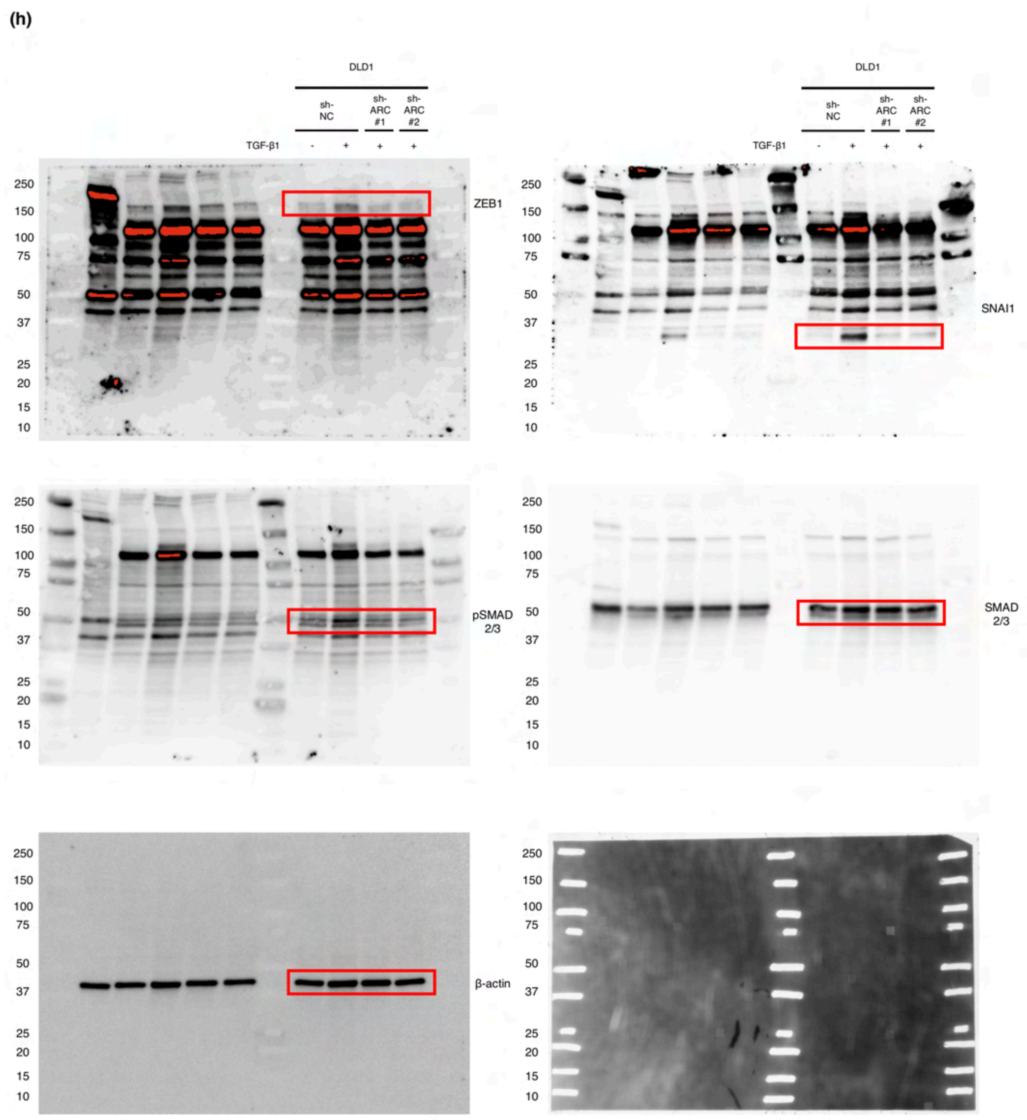

Supplementary Fig. 11: Full length blots of figures. (a) Full length blots of Figure 3c. Results of ZEB1 were shown from different membranes using the same samples. A neuroblastoma cell line, N2a, was used as a positive control for ZEB1 and ARC. (b) Full length blots of Supplementary Figure 6. (c) Full length blots of Supplementary Figure 8a. (d) Full length blots of Figure 5b. The membrane was cropped before hybridization with anti-ARC antibody, anti-pSMAD2/3 antibody, and anti-SMAD2/3 antibody. (e) Full length blots of Supplementary Figure 9b. (f) Full length blots of Figure 5c. (g) Full length blots of Supplementary Figure 9c. (h) Full length blots of Supplementary Figure 9d.

Red boxes indicate the region used in the figures.

Supplementary Table

Supplementary Table 1. Additional patient characteristics according to ARC expression

Factors	ARC expression	
	Negative (n=71)	Positive (n=29)
Primary tumor site (C/A/T/D/S/R)	3/14/4/5/24/21	1/3/1/1/15/8
Histological grade (Tub1/Tub2/Por/Muc)	33/33/2/3	13/16/0/0
Tumor invasion (Tis/T1/T2/T3/T4a/T4b)	5/13/13/36/2/2	2/4/3/18/1/1
Lymph node metastasis (N0/N1a/N1b/N1c/N2a/N2b)	52/9/6/0/2/2	15/6/6/0/1/1
Pathological stage (0/I/IIA/IIB/IIIA/IIIB/IIIC)	5/23/22/0/2/3/14/2	2/5/7/0/1/2/11/1

Supplementary Text 1: Previous reports of candidate genes on EMT, cell invasion, or migration (Fig. 2a).

ADAM21 promotes invasion, migration, and metastasis in HCC[51]. Circular RNA ANKS1B promotes breast cancer invasion and metastasis both in vitro and in vivo by inducing EMT[52]. A small-molecule inhibitor that blocked the AURKC-IκBα interaction significantly inhibits MDA-MB-231 cell invasion, migration, and colony formation[53]. BCAM impairs adhesion of KRAS-mutant CRC cells to endothelial cells and promotes metastasis[54]. BEST1 promotes EMT of renal collecting duct cells[55]. MicroRNA-495-3p inhibits the S1PR3 (C9orf47) /SMAD2/3 pathway and suppresses the EMT process in pulmonary fibrosis[56]. The CaMK2-specific inhibitor KN93 prohibits HCT116 cell migration and invasion[57]. DPCR1 promotes proliferation, migration, and invasion in pancreatic ductal adenocarcinoma[58]. FABP4 promotes EMT in cervical squamous cell carcinoma through AKT/GSK3β/Snail signaling pathway[59]. The long non-coding RNA FLJ33360 promotes migratory and invasive ability in HCC by targeting miR-140/MMP9[60]. FLT4/VEGFR3 mediates TGFb1-induced EMT in NSCLC[61]. FOXS1promotes EMT in gastric cancer[62]. The long non-coding RNA HOTAIR promotes EMT in CRC[63]. HOXC10 promotes EMT in oral SCC[64]. HOXC13-AS upregulates HOXC13 expression and promotes EMT[65]. HOXC6 promotes EMT in HCC[29], oral squamous

cell carcinoma[30], and cervical cancer[31]. MicroRNA-377 regulates IRX3 and promotes migration and invasion in HCC[66]. KLF17 induces EMT via direct activation of TWIST1 in endometrioid endometrial cancer[67]. LEP induced EMT in breast cancer[68]. MARK4 inhibits the Hippo pathway and promotes migration in breast cancer[32]. NOVA1 promotes EMT in breast cancer[69] and gastric cancer[70]. NRSN2 promotes migration and invasion in breast cancer[71]. NTF4 promotes migration and invasion in CRC[72]. NTSR1 promotes migration in CRC cell lines[73]. Blocking expression of PANX2 results in suppression of proliferation, migration, and invasion in prostate cancer cells[74]. PIPKIc90 is required for the migration and invasion of MDA-MB-231 human breast cancer cells and HeLa human cervical cancer cells[75]. Knockdown of NFATC3-PLA2G15 reduced mRNA expression of EMT markers[76]. Depletion of PPP1R1A results in a significant decrease in oncogenic transformation and cell migration in Ewing sarcoma[77]. RNF216 promotes CRC cell proliferation and migration in vitro and in vivo[78]. Ectopic overexpression of SOX18 causes EMT in high-grade serous ovarian cancers[79]. ST8SIA3 synthesizes A2B5 epitope, which promotes proliferation, migration, clonogenicity, and tumorigenesis[80].

EMT: epithelial-mesenchymal transition; CRC: colorectal cancer; HCC: hepatocellular carcinoma

Supplementary Text 2: Programmed cell death protein 1 (PDCD1) is an immune-inhibitory checkpoint expressed on activated T cells. CD274 encodes PDCD1's ligand, PD-L1, and interaction of this ligand with the receptor inhibits T-cell functions[81]. Cytotoxic T-lymphocyte-associated protein 4 (CTLA4) is also an immune-inhibitory checkpoint expressed on activated T cells and regulatory T cells. Interaction of CTLA4 and its ligand, B7, regulates T-cell activation[81]. β2-microglobulin (B2M) is a component of class I major histocompatibility complex, and its loss or mutation is involved in tumor immune evasion[82].

Supplementary References

51. Honda, H. *et al.* Overexpression of a disintegrin and metalloproteinase 21 is associated with motility, metastasis, and poor prognosis in hepatocellular carcinoma. *Sci. Rep.* **7**, 15485 (2017).

52. Zeng, K. *et al.* The pro-metastasis effect of circANKS1B in breast cancer. *Mol. Cancer* **17**, 160 (2018).

53. Han, E. H. *et al.* A small-molecule inhibitor targeting the AURKC-IκBα interaction decreases transformed growth of MDA-MB-231 breast cancer cells. *Oncotarget* **8**, 69691–69708 (2017).

54. Bartolini, A. *et al.* BCAM and LAMA5 mediate the recognition between tumor cells and the endothelium in the metastatic spreading of KRAS-mutant colorectal cancer. *Clin. Cancer Res.* **22**, 4923–4933 (2016).

55. Aldehni, F. *et al.* Bestrophin 1 promotes epithelial-to-mesenchymal transition of renal collecting duct cells. *J. Am. Soc. Nephrol. JASN* **20**, 1556–1564 (2009).

56. Gong, L. *et al.* S1PR3 deficiency alleviates radiation-induced pulmonary fibrosis through the regulation of epithelial–mesenchymal transition by targeting miR-495-3p. *J. Cell. Physiol.* **235**, 2310–2324 (2020).

57. Chen, W. *et al.* Ca2+/calmodulin-dependent protein kinase II regulates colon cancer proliferation and migration via ERK1/2 and p38 pathways. *World J. Gastroenterol.* **23**, 6111–6118 (2017).

58. Yan, J. *et al.* High expression of diffuse panbronchiolitis critical region 1 gene promotes cell proliferation, migration and invasion in pancreatic ductal adenocarcinoma. *Biochem. Biophys. Res. Commun.* **495**, 1908–1914 (2018).

59. Jin, J. *et al.* Fatty acid binding protein 4 promotes epithelial-mesenchymal transition in cervical squamous cell carcinoma through AKT/GSK3β/Snail signaling pathway. *Mol. Cell. Endocrinol.* **461**, 155–164 (2018).

60. Lu, Z. *et al.* LncRNA FLJ33360 accelerates the metastasis in hepatocellular carcinoma by targeting miRNA-140/MMP9 axis. *Am. J. Transl. Res.* **12**, 583–591 (2020).

61. Duan, L. *et al.* VEGFC/VEGFR3 axis mediates TGFβ1-induced epithelial-to-mesenchymal transition in non-small cell lung cancer cells. *PLoS ONE* **13**, (2018).

62. Wang, S. *et al.* FOXS1 is regulated by GLI1 and miR-125a-5p and promotes cell proliferation and EMT in gastric cancer. *Sci. Rep.* **9**, (2019).

63. Dou, J. *et al.* Decreasing lncRNA HOTAIR expression inhibits human colorectal cancer stem cells. *Am. J. Transl. Res.* **8**, 98–108 (2016).

64. Dai, B.-W. *et al.* HOXC10 promotes migration and invasion via the WNT-EMT signaling pathway in oral squamous cell carcinoma. *J. Cancer* **10**, 4540–4551 (2019).

65. Li, W. *et al.* HOXC13-AS accelerates cell proliferation and migration in oral squamous cell carcinoma via miR-378g/HOXC13 axis. *Oral Oncol.* **111**, 104946 (2020).

66. Wang, P., Zhuang, C., Huang, D. & Xu, K. Downregulation of miR-377 contributes to IRX3 deregulation in hepatocellular carcinoma. *Oncol. Rep.* **36**, 247–252 (2016).

67. Dong, P. *et al.* Identification of KLF17 as a novel epithelial to mesenchymal transition inducer via direct activation of TWIST1 in endometrioid endometrial cancer. *Carcinogenesis* **35**, 760–768 (2014).

68. Yan, D., Avtanski, D., Saxena, N. K. & Sharma, D. Leptin-induced epithelial-mesenchymal transition in breast cancer cells requires β-catenin activation via Akt/GSK3- and MTA1/Wnt1 protein-dependent pathways. *J. Biol. Chem.* **287**, 8598–8612 (2012).

69. Tang, S. *et al.* Identification of NOVA family proteins as novel β-catenin RNA-binding proteins that promote epithelial-mesenchymal transition. *RNA Biol.* **17**, 881–891 (2020).

70. Li, K., Zhu, X., Chen, X. & Wang, X. MicroRNA-27a-3p promotes epithelial-mesenchymal transition by targeting NOVA alternative splicing regulator 1 in gastric cancer. *Mol. Med. Rep.* **21**, 1615–1622 (2020).

71. Ren, F., Zhang, W., Lu, S., Ren, H. & Guo, Y. NRSN2 promotes breast cancer metastasis by activating PI3K/AKT/mTOR and NF-κB signaling pathways. *Oncol. Lett.* **19**, 813–823 (2020).

72. Yang, Z. *et al.* Upregulated NTF4 in colorectal cancer promotes tumor development via regulating autophagy. *Int. J. Oncol.* **56**, 1442–1454 (2020).

73. Kim, J. T., Weiss, H. L. & Evers, B. M. Diverse expression patterns and tumorigenic role of neurotensin signaling components in colorectal cancer cells. *Int. J. Oncol.* **50**, 2200–2206 (2017).

74. Liao, D. *et al.* Identification of Pannexin 2 as a novel marker correlating with ferroptosis and malignant phenotypes of prostate cancer cells. *OncoTargets Ther.* **13**, 4411–4421 (2020).

75. Li, X. *et al.* Ubiquitylation of phosphatidylinositol 4-phosphate 5-kinase type I γ by HECTD1 regulates focal adhesion dynamics and cell migration. *J. Cell Sci.* **126**, 2617–2628 (2013).

76. Jang, J.-E. *et al.* NFATC3–PLA2G15 fusion transcript identified by RNA sequencing promotes tumor invasion and proliferation in colorectal cancer cell lines. *Cancer Res. Treat. Off. J. Korean Cancer Assoc.* **51**, 391–401 (2019).

77. Luo, W. *et al.* Protein phosphatase 1 regulatory subunit 1A in ewing sarcoma tumorigenesis and metastasis. *Oncogene* **37**, 798–809 (2018).

78. Wang, H. *et al.* RNF216 contributes to proliferation and migration of colorectal cancer via suppressing BECN1-dependent autophagy. *Oncotarget* **7**, 51174–51183 (2016).

79. Lawrenson, K. *et al.* A Study of high-grade serous ovarian cancer origins implicates the SOX18 transcription factor in tumor development. *Cell Rep.* **29**, 3726-3735.e4 (2019).

80. Baeza-Kallee, N. *et al.* Glycolipids recognized by A2B5 antibody promote proliferation, migration, and clonogenicity in glioblastoma cells. *Cancers* **11**, (2019).

81. Duraiswamy, J., Kaluza, K. M., Freeman, G. J. & Coukos, G. Dual blockade of PD-1 and CTLA-4 combined with tumor vaccine effectively restores T cell rejection function in tumors. *Cancer Res.* **73**, 3591–3603 (2013).

82. Restifo, N. P. *et al.* Loss of functional beta2-microglobulin in metastatic melanomas from five patients receiving immunotherapy. *J. Natl. Cancer Inst.* **88**, 100–108 (1996).
